# Incidence of Lyme Borreliosis in Europe: A Systematic Review (2005–2020)

**DOI:** 10.1089/vbz.2022.0070

**Published:** 2023-04-12

**Authors:** Leah Burn, Andrew Vyse, Andreas Pilz, Thao Mai Phuong Tran, Mark A. Fletcher, Frederick J. Angulo, Bradford D. Gessner, Jennifer C. Moïsi, James H. Stark

**Affiliations:** ^1^ P95 Pharmacovigilance and Epidemiology, Princeton, New Jersey, USA.; ^2^ Pfizer Vaccines Medical, Walton Oaks, United Kingdom.; ^3^ Pfizer Global Medical Affairs, Vaccines, Vienna, Austria.; ^4^ P95 Pharmacovigilance and Epidemiology, Leuven, Belgium.; ^5^ Pfizer Emerging Markets Medical Affairs, Vaccines, Paris, France.; ^6^ ^,i^Vaccines Medical Development, Scientific and Clinical Affairs, Pfizer, Inc., Collegeville, Pennsylvania, USA.; ^7^ Pfizer Medical Development, Scientific and Clinical Affairs, Vaccines, Paris, France.

**Keywords:** Lyme Borreliosis, incidence, epidemiology, Europe, systematic review

## Abstract

**Background:**

Lyme borreliosis (LB) is the most common tick-borne disease in Europe, but the burden of disease is incompletely described.

**Methods:**

We conducted a systematic review across PubMed, EMBASE, and CABI Direct (Global Health) databases, from January 1, 2005, to November 20, 2020, of epidemiological studies reporting incidence of LB in Europe (PROSPERO, CRD42021236906).

**Results:**

The systematic review yielded 61 unique articles describing LB incidence (national or subnational) in 25 European countries. Substantial heterogeneity in study designs, populations sampled, and case definitions restricted data comparability. The European Union Concerted Action on Lyme Borreliosis (EUCALB)–published standardized LB case definitions were used by only 13 (21%) of the 61 articles. There were 33 studies that provided national-level LB incidence estimates for 20 countries. Subnational LB incidence was available from an additional four countries (Italy, Lithuania, Norway, and Spain). The highest LB incidences (>100 cases per 100,000 population per year [PPY]) were reported in Belgium, Finland, the Netherlands, and Switzerland. Incidences were 20–40/100,000 PPY in the Czech Republic, Germany, Poland, and Scotland and <20/100,000 PPY in Belarus, Croatia, Denmark, France, Ireland, Portugal, Russia, Slovakia, Sweden, and the United Kingdom (England, Northern Ireland, and Wales); markedly higher incidences were observed at the subnational level (up to 464/100,000 PPY in specific local areas).

**Conclusions:**

Although countries in Northern (Finland) and Western (Belgium, the Netherlands, and Switzerland) Europe reported the highest LB incidences, high incidences also were reported in some Eastern European countries. There was substantial subnational variation in incidence, including high incidences in some areas of countries with low overall incidence. This review, complemented by the incidence surveillance article, provides a comprehensive view into LB disease burden across Europe that may guide future preventive and therapeutic strategies—including new strategies on the horizon.

## Introduction

Lyme borreliosis (LB) is a tick-borne zoonosis caused by different genospecies of *Borrelia burgdorferi sensu lato* (*Bbsl*) complex (Estrada-Pena et al., 2018, Margos et al., 2009, Radolf et al., 2021, Rauter and Hartung, 2005, Richter and Matuschka, 2006, Stanek and Strle, 2018, Stanek et al., 2012, Strnad et al., 2017, Woitzik and Linder, 2021, Wolcott et al., 2021). In 2006, the World Health Organization (WHO) estimated that 85,000 cases of LB occur annually in the 25 countries of the European Union, although it is likely that there is substantial underestimation of this disease burden due to lack of clinical awareness, insensitive laboratory diagnostics, and incomplete reporting (Lindgren and Jaenson, 2006).

Geographic variations in LB disease burden reflect likelihood of exposure to infected ticks. Thus, the incidence of LB is higher in areas where there is an abundance of vertebrate animals that serve as reservoirs for infected ticks (Lindgren and Jaenson, 2006). LB is also more common in persons who undertake outdoor occupations or leisure activities that increase the risk of exposure to tick bites in *Bbsl*-endemic areas (Magnavita et al., 2022).

After infection of *Bbsl*, spirochetes burrow between tissues, bones, cells, joints, and nerves, and they can cross the blood–brain barrier into the central nervous system. There are various clinical manifestations of disease (Kullberg et al., 2020, Lantos et al., 2021, Marques et al., 2021, Stanek et al., 2011). Within days to weeks, *Bbsl* disseminates from the tick bite site to other body regions causing early localized infection and often—although not always—erythema migrans (EM) (Steere et al., 2016). Sometimes, Borrelial lymphocytoma also develops. After weeks or months, *Bbsl* uses a chemotaxis machinery system to disseminate into the host, causing early disseminated LB or late disseminated LB. Clinical manifestations of the disseminate form of the disease include: Lyme neuroborreliosis (LNB), Lyme carditis (LC), Lyme arthritis (LA), and acrodermatitis chronica atrophicans (ACA) (Aucott et al., 2009, Bernard et al., 2019, Stanek and Strle, 2018, Stanek et al., 2012, Steere et al., 2016, Verhaegh et al., 2017).

Seroconversion can occur with or without clinical symptoms (Kullberg et al., 2020, Marques et al., 2021). A diagnosis of LB is first assessed clinically (Stanek and Strle, 2018, Stanek et al., 2012, Steere et al., 2016). After clinical evaluation, LB diagnosis is often supported through laboratory testing that can include serology, including detection of specific intrathecal antibodies, and microbiological examination of infected tissue in patients with suspected LNB. Serum antibody tests include enzyme immunoassays, immunofluorescence assays, enzyme-linked immunosorbent assays (ELISAs), or Western blots, and combinations of these. These tests vary in sensitivity and specificity, which can impact the likelihood of diagnosis of LB, and this has wide consequences for studies of LB epidemiology; antibody detection is not always the equivalent of disease (Kodym et al., 2018, Leeflang et al., 2016).

The transient presentation of EM, the broad spectrum of clinical presentations observed in patients with early and late disseminated disease, and complexity of serological testing have impeded the development of generally accepted standard case definitions for LB, both in public health surveillance and in epidemiological studies (Stanek and Strle, 2018, Stanek et al., 2012). In the absence of widely accepted standardized case definitions for LB in Europe, the European Union Concerted Action on Lyme Borreliosis (EUCALB) published case definitions for manifestations of LB in 1996 and updated them in 2011 in an effort to encourage Europe-wide implementation (Stanek et al., 2011). To date, however, very few LB surveillance systems in Europe implement the EUCALB definitions (also see Nagarajan et al., in this edition).

The European Centre for Disease Prevention and Control (ECDC) has employed a reportable LNB case definition to detect and monitor LNB cases in Europe (European Centre for Disease Prevention and Control, 2018; European Commission, 2018; The Lancet, 2018), yet how often it is utilized across countries remains unknown. LNB is a clinical manifestation that represents the more severe, disseminated form of the disease involving systemic involvement (Radolf et al., 2021, Rauer et al., 2018, Trevisan et al., 2020). It is an appropriate indicator for surveillance given its high specificity in diagnosis and reproducibility in measurement (Stanek and Strle, 2018; The Lancet, 2018; Van den Wijngaard et al., 2017).

With EM as the leading indicator, and LNB to measure more severe forms of the disease, surveillance of other clinical manifestations periodically could also add value to surveillance and epidemiological studies to provide full insights of LB epidemiology in Europe (Van den Wijngaard et al., 2017). Understanding the population-based incidence of LB is important for targeting and evaluating LB prevention strategies, which could potentially include vaccination. Because of the inherent limitations in LB surveillance (Stanek et al., 2011, Van den Wijngaard et al., 2017; and Nagarajan et al., in this edition), more complete epidemiological studies may provide complementary data to understand LB incidence. Recent systematic reviews of LB have attempted to quantify LB incidence but have been limited to Western Europe (Sykes, 2014, Vandekerckhove et al., 2021). We conducted a comprehensive systematic review across all of Europe to understand the national- and subnational-level incidence of LB reported in the published literature over the past 15+ years.

## Methods

The methodology, search strategy, and inclusion and exclusion criteria for the systematic review and analysis are included in a protocol developed by the Lyme Review Group, which included experts in Lyme vaccine development, clinical epidemiologists, and statisticians. The protocol was based on Preferred Reporting Items for Systematic Reviews and Meta-Analyses (PRISMA, 2020) guidelines and was registered in PROSPERO (CRD42021236906). The protocol is for multi-objective review for a global study on LB. For the purposes of this article, we focus our scope on incidence estimates of LB in Europe.

### Search strategy

We conducted a multi-database systematic review across CABI Direct (Global Health), EMBASE, and PubMed databases, with no restrictions on language, from January 1, 2005, to November 20, 2020, using the following search terms: *Lyme*, *Borrelia*, and *borreliosis*.

All citations were merged into a database, and duplicates were removed. Titles and abstracts were screened independently by two reviewers for their relevance to the study objectives. Selected full-text articles were assessed based on predefined inclusion and exclusion criteria, by two reviewers. Full-text articles published in other European languages were translated into English using *DeepL* (DeepL SE, 2022). For articles that were not easily translated in *DeepL*, such as Finnish, we utilized P95/Pfizer colleagues who were native speakers or fluent in these languages to check and elaborate on translation accuracy. Relevant variables from selected articles were extracted into *DistillerSR* (Evidence Partners, 2021). A reviewer independently checked 20% of the articles and their extractions. All discrepancies identified during each phase were discussed and resolved.

### Inclusion and exclusion criteria

We selected articles (obtained from our search) reporting LB incidence and/or LB cases for this study. Health-economic or cost studies, case studies, animal studies, as well as studies of biomedical mechanisms, modeling or simulations, or management or diagnostic guidelines, were excluded. Data only available in abstract form from conferences, letters, perspective or opinion papers, or commentaries were also excluded. Review articles were not included but were scanned for references. Articles reporting the results of national surveillance were excluded if the data were duplicated from the available public health surveillance reports, which have been analyzed and published separately (Burn et al., 2023, in this issue).

### Analysis

We synthesized data, with relevant descriptive and key outcome variables, into tables (Campbell et al., 2020). For the purposes of data presentation, we considered four European regions per the WHO Regional Classification scheme and organized national data accordingly (Table 1) (World Health Organization, 2022).

**Table 1.** Countries with Published National and/or Subnational Estimates of Lyme Borreliosis Cases or Incidence

European region	Countries
Eastern Europe	**Belarus**, **Czech Republic**, Hungary,^a^**Poland**, Romania, **Russia**, **Slovakia**, Slovenia^a^
Northern Europe	
Baltic states	Lithuania
Nordic region	**Denmark**, **Finland**, Norway, **Sweden**
The United Kingdom and Ireland	**England**, **Ireland**, **Northern Ireland**, **Scotland**, **Wales**
Southern Europe	**Croatia**, Italy, **Portugal**, Spain
Western Europe	**Belgium**, **France**, **Germany**, **The Netherlands**, **Switzerland**

Countries with national estimates are indicated in bold (data provided in Tables 2 − 6).

^a^ There were publications that reported LB cases in Hungary and Slovenia but not incidence estimates (Supplementary Table S7).

LB, Lyme borreliosis.

*Source:* World Health Organization (2022).

Forest plots were produced for *national-level* incidence estimates, organized by European region, by country, and study characteristics (study period, data source, and case definitions). The 95% confidence intervals (CIs) were displayed as applicable. When a study reported the cumulative number of cases over a study period without reporting the corresponding average number of cases, we calculated the mean number of cases by dividing the cumulative number of cases over the year period. In the case that the 95% CI of the LB incidence was not reported, we calculated it using the exact binomial method (Wilson, 1927), given the availability of the number of cases (and/or the corresponding sample sizes) and the LB incidence.

Plots were further stratified by clinical manifestations when reported in the study. Point estimates for each study are represented by a black box, and the magnitude of the black box represents the size of the study. The 95% CIs are represented by the horizontal lines for each plot. We measured the heterogeneity of the data using the *I*^2^ statistic (Campbell et al., 2020, Deeks et al., 2022, Higgins et al., 2003). Meta-analyses were performed, but the results are not reported due to the considerable heterogeneity of the data. All analyses were performed using the statistical software R (RStudio, version 1.4.1103) (R Core Team, 2021; RStudio Team, 2020).

## Results

### Search results

**FIG. 1.**
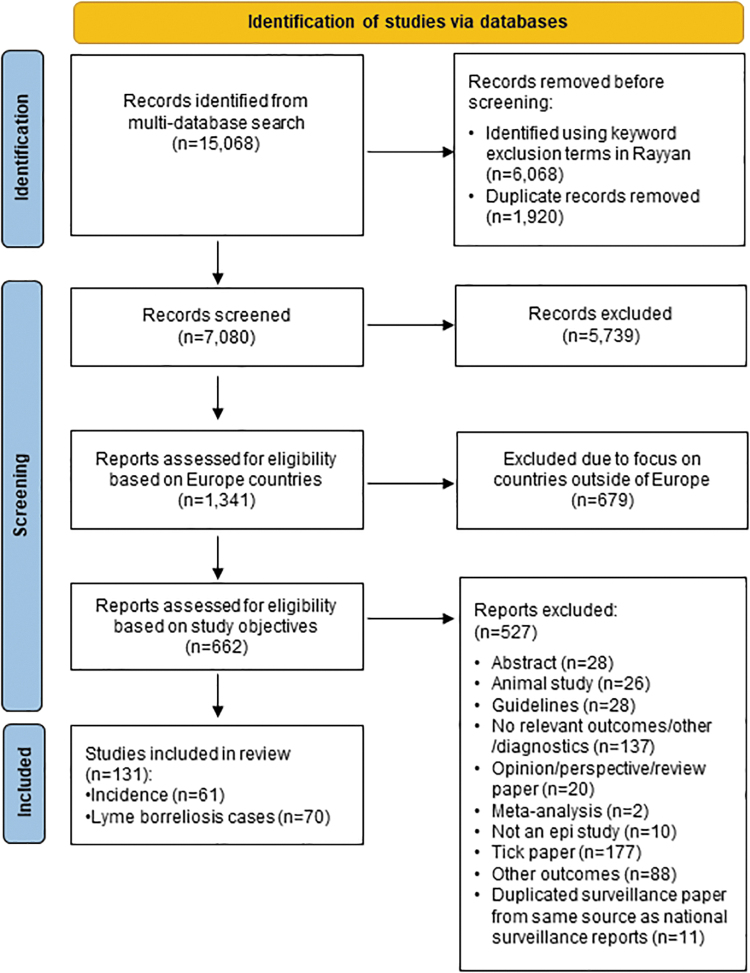
PRISMA flow diagram. *n*, number of articles; PRISMA, Preferred Reporting Items for Systematic Reviews and Meta-Analyses.

The systematic review captured 72 articles that reported LB incidence estimates, of which 11 duplicated data already provided by the national surveillance reports (provided in our companion incidence surveillance article for Europe, Burn et al., 2023, in this issue). This yielded 61 unique articles describing LB incidence (national or subnational) in 25 countries (Fig. 1). No published data were obtained for Albania, Andorra, Austria, Bosnia and Herzegovina, Greece, Iceland, Liechtenstein, Luxembourg, Malta, Moldova, Monaco, Montenegro, North Macedonia, San Marino, or Ukraine.

Among the studies, there were countries with national LB incidence (*n* = 28); countries reported subnational LB incidence estimates (*n* = 11; with overlap of both national and subnational estimates from some studies) and subnational (*n* = 33) (Fig. 1). Estimates of national LB incidence are presented in Tables 2–6; subnational data are provided in Supplementary Tables S1−S6. Subnational variations in incidence values were substantial in many countries. We illustrate examples of this in the text below, and the reader is referred to Supplementary Tables S7–S11 for a more in-depth review.

**Table 2.** Estimates of National Incidence of Lyme Borreliosis (Cases per 100,000 Population per Year) in Eastern Europe from the Literature Published from 2005 to 2020

Country	Data source	Study design	Study population	Case definition	Study period	No. of cases	Incidence (cases per 100,000 PPY)
Belarus (Karaban et al., 2009)	Regional sanitary and epidemiological data	Retrospective cohort	National population	Clinical and history of tick bites	1998		1.3
					1999		1.0
					2000		1.9
					2001		1.8
					2002		1.8
					2003		5.1
					2004		5.3
					2005		5.4
					2006		9.1
					2007		6.7
Czech Republic (Kriz et al., 2018)	National Reference Laboratory for Lyme Borreliosis, National Reference Laboratory for Arboviruses	Retrospective, surveillance cohort	National population	Legislated definition	2007 − 2016	39,074 (LB), 24,263 (EM), 9791 (LNB), 37 (LC)	37.3
Czech Republic and Poland border region (Stefanoff et al., 2014)	Central Statistical Office of Poland	Retrospective, observational	National population	All cases reported by physicians	1999–2008	6783	
					1999		1.0
					2000		3.4
					2001		5.9
					2002		4.1
					2003		9.7
					2004		9.2
					2005		13.0
					2006		19.5
					2007		21.2
					2008		25.1
Russia (Kovalenko et al., 2012)	Notification data and laboratory results	Observational, retrospective, surveillance cohort	Eighteen administrative areas, population 684,000	None given	2002		3.3
					2010	36	4.2
					2011	58	8.5
Slovakia (Svihrova et al., 2011)	Epidemiological Informative System of Communicable Diseases of the Slovak Republic	Retrospective cohort	National population	EM	1999 − 2008	5435	10.1
					1999		8.8
					2008		14.6
				Early disseminated infection	1999 − 2008	506	0.9
				Late, chronic persistent infection	1999 − 2008	1408	2.6
					1999		1.7
					2007		4.4
					2008		3.7

Incidence estimates and 95% CIs have been rounded to the first decimal to the right for consistent level of precision.

CI, confidence interval; EM, erythema migrans; LC, Lyme carditis; LNB, Lyme neuroborreliosis; PPY, population per year.

**Table 3.** Estimates of National Incidence of Lyme Borreliosis (Cases per 100,000 Population per Year) in Northern Europe (Nordic Region) from the Literature Published from 2005 to 2020

Country	Data source	Study design	Study population	Case definition	Study period	No. of cases	Incidence (cases per 100,000 PPY)
Denmark (Dessau et al., 2015)	Danish Notification System for Infectious Diseases, Danish Microbiology Database, Danish civil registration system	Prospective cohort	National population	LNB	2010 − 2012	533	3.2
Denmark (Tetens et al., 2020)	Danish national registries	Retrospective cohort	National population	LNB—positive for intrathecal antibodies	1996 − 1999	480	2.2
					2000 − 2003	579	2.7
					2004 − 2007	714	3.3
					2008 − 2011	616	2.8
					2012 − 2015	402	1.8
					1996 − 2015	2791	2.6
Finland (Sajanti et al., 2017)	National infectious disease register, national hospital discharge register, register for primary health care visits	Retrospective cohort	National population	Microbiologically confirmed	1995	345	7
					2014	1679	31
				Clinically diagnosed	2011–2014	11,793	
					2011		44
					2014		61
				Estimated annual	2011	5011	93
				Estimated annual	2014	6440	118
Sweden (Dahl et al., 2019)	National laboratory reporting	Retrospective cohort	National population	LNB—positive CSF fluid-serum antibody index	2010	578	6.1
					2011	689	7.3
					2012	559	5.8
					2013	561	5.9
					2014	604	6.3

Incidence estimates and 95% CIs have been rounded to the first decimal to the right for consistent level of precision.

CI, confidence interval; CSF, cerebrospinal fluid.

**Table 4.** Estimates of National Incidence of Lyme Borreliosis (Cases per 100,000 Population per Year) in Northern Europe (the United Kingdom and Ireland) from the Literature Published from 2005 to 2020

Country	Data source	Study design	Study population	Case definition	Study period	No. of cases	Incidence (cases per 100,000 PPY) (95% CI)
England, Wales (Tulloch et al., 2019b)	PHE RIPL LIMS, laboratory-confirmed cases	Prospective ecological	National population	Serological diagnosis	2013–2016	3986	
					2013		1.6
					2016		2.0
England, Wales (Tulloch et al., 2019a)	Hospital episode statistics (England); patient episode database (Wales)	Retrospective cohort	Hospitalized patients with LB	ICD-10 codes	1998–2005	2361	
					1998		0.08
					2005		0.53
Ireland (Forde et al., 2021)	Laboratory records	Retrospective cohort	National population 2 − 18 years of age	LB NICE guidelines^a^	2012 − 2016	63 (LB), 27 (EM and/or influenza-like symptoms), 1 (LA)	1.15
Scotland (Mavin et al., 2015)	National Lyme Borreliosis Testing Laboratory and questionnaires from all laboratory-confirmed cases within NHS Highland	Retrospective cohort, seroepidemiological	National population	ELISA (IgM/IgG), then immunoblot	2008 − 2013	1865	6.8
					2008	339	7.8
					2009	393	9
					2010	440	9.8
					2011	308	6.7
					2012	210	4.1
					2013	175	3.1
United Kingdom (Cairns et al., 2019)	CPRD	Retrospective cohort	Eight percent of national population	Read codes for LB, suspected and possible LB	2001–2012	4083	
					2001	60	1.6 (1.2 − 2)^b^
					2002	115	2.9 (2.3 − 3.4)
					2003	90	2.1 (1.6 − 2.5)
					2004	161	3.6 (3 − 4.1)
					2005	211	4.5 (3.9 − 5.1)
					2006	314	6.3 (5.6 − 7.1)
					2007	422	8.6 (7.7 − 9.4)
					2008	445	9.0 (8.1 − 9.8)
					2009	538	10.9 (9.9 − 11.9)
					2010	564	11.5 (10.5 − 12.5)
					2011	568	11.9 (10.9 − 12.9)
					2012	595	12.1 (11.1 − 13.2)
			Scotland		2010 − 2012	526	37.3 (34.2 − 40.7)
			Wales		2010 − 2012	71	6.0 (4.6 − 7.6)
United Kingdom (Tulloch et al., 2020)	THIN	Retrospective cohort	Six percent of national population	Read codes specific to LB, suspected LB, or related conditions		3725	
					1998	1035	1.8 (1.4 − 2.3)
					1999	1015	1.7 (1.4 − 2.2)
					2000	1195	2.0 (1.7 − 2.5)
					2001	1141	1.9 (1.6 − 2.4)
					2002	1698	2.9 (2.4 − 3.4)
					2003	1300	2.2 (1.8 − 2.6)
					2004	1733	2.9 (2.5 − 3.4)
					2005	1951	3.2 (2.8 − 3.7)
					2006	2147	3.5 (3.1 − 4.0)
					2007	2404	3.9 (3.5 − 4.4)
					2008	2634	4.3 (3.8 − 4.8)
					2009	2802	4.5 (4.0 − 5.0)
					2010	2655	4.2 (3.8 − 4.8)
					2011	2639	4.2 (3.7 − 4.7)
					2012	2389	3.8 (3.3 − 4.2)
					2013	2859	4.5 (4.0 − 5.0)
					2014	2170	3.4 (2.9 − 3.9)
					2015	3562	5.5 (4.9 − 6.1)
					2016	3210	4.9 (4.3 − 5.6)
			England		1998		1.6 (1.1 − 2.1)
					1999		1.5 (1.13 − 2.0)
					2000		1.9 (1.4 − 2.4)
					2001		1.8 (1.4 − 2.3)
					2002		2.3 (1.9 − 2.9)
					2003		2.1 (1.7 − 2.6)
					2004		2.7 (2.3 − 3.3)
					2005		2.5 (2.0 − 3.0)
					2006		3.0 (2.5 − 3.5)
					2007		3.3 (2.8 − 3.9)
					2008		3.6 (3.1 − 4.2)
					2009		3.7 (3.2 − 4.3)
					2010		3.4 (2.8 − 4.0)
					2011		3.2 (2.7 − 3.7)
					2012		3.2 (2.7 − 3.7)
					2013		3.4 (2.9 − 4.0)
					2014		2.7 (2.2 − 3.2)
					2015		4.1 (3.4 − 4.8)
					2016		3.3 (2.6 − 4.1)
			Northern Ireland		1998		0 (0 − 1.6)
					1999		0 (0 − 2.7)
					2000		0 (0 − 1.2)
					2001		0.5 (0 − 2.2)
					2002		0.9 (0.2 − 2.9)
					2003		0 (0 − 1.1)
					2004		0.4 (0 − 1.9)
					2005		0.8 (0.2 − 2.5)
					2006		1.1 (0.3 − 3.0)
					2007		1.7 (0.7 − 3.8)
					2008		1.4 (0.5 − 3.4)
					2009		1.1 (0.3 − 2.8)
					2010		0.7 (0.1 − 2.2)
					2011		1.0 (0.3 − 2.7)
					2012		2.0 (0.8 − 4.2)
					2013		1.3 (0.4 − 3.2)
					2014		1.0 (0.3 − 2.6)
					2015		2.3 (1.0 − 4.5)
					2016		1.0 (0.3 − 2.6)
			Scotland		1998		5.2 (2.9 − 8.7)
					1999		4.7 (2.8 − 7.4)
					2000		4.2 (2.8 − 5.9)
					2001		3.1 (2.2 − 4.5)
					2002		5.7 (4.2 − 7.5)
					2003		4.2 (3.0 − 5.7)
					2004		5.6 (4.2 − 7.3)
					2005		8.6 (6.9 − 10.7)
					2006		8.2 (6.5 − 10.2)
					2007		9.0 (7.3 − 11.1)
					2008		10.1 (8.3 − 12.2)
					2009		11.0 (9.1 − 13.2)
					2010		10.1 (8.3 − 12.1)
					2011		11.4 (9.5 − 13.6)
					2012		8.2 (6.6 − 10.0)
					2013		11.0 (9.2 − 13.1)
					2014		8.3 (6.7 − 10.1)
					2015		12.7 (10.7 − 14.9)
					2016		10.7 (8.9 − 12.8)
			Wales		1998		1.6 (0.5 − 3.8)
					1999		1.1 (0.3 − 2.9)
					2000		0.9 (0.2 − 2.3)
					2001		1.3 (0.5 − 2.9)
					2002		3.0 (1.7 − 5.1)
					2003		0.6 (0.2 − 1.6)
					2004		0.9 (0.3 − 2.0)
					2005		1.4 (0.7 − 2.6)
					2006		1.3 (0.6 − 2.5)
					2007		1.4 (0.7 − 2.6)
					2008		0.9 (0.4 − 1.9)
					2009		1.5 (0.8 − 2.6)
					2010		2.4 (1.5 − 3.8)
					2011		1.1 (0.5 − 2.1)
					2012		1.4 (0.7 − 2.4)
					2013		2.0 (1.2 − 3.2)
					2014		0.7 (0.2 − 1.4)
					2015		1.8 (1.0 − 2.9)
					2016		2.5 (1.6 − 3.9)

Incidence estimates and 95% CIs have been rounded to the first decimal to the right for consistent level of precision.

^a^ Available at https://www.nice.org.uk/guidance/ng95

^b^ Incidence (person-time) reported, rather than incidence proportion.

CI, confidence interval; CPRD, Clinical Practice Research Datalink; ELISA, enzyme-linked immunosorbent assay; ICD-10, International Classification of Diseases version 10; IgM/IgG, immunoglobulin M/immunoglobulin G; LA, Lyme arthritis; LIMS, laboratory information management system; NHS, National Health Service; NICE, National Institute for Health and Care Excellence; PHE RIPL, Public Health England Rare and Imported Pathogens Laboratory; PPY, population per year; THIN, The Health Improvement Network.

**Table 5.** Estimates of National Incidence of Lyme Borreliosis (Cases per 100,000 Population per Year) in Southern Europe from the Literature Published from 2005 to 2020

Country	Data source	Study design	Study population	Case definition	Study period	No. of cases	Incidence (cases per 100,000 PPY)
Croatia (Mulić et al., 2011)	Croatian Institute for Public Health, obtained through mandatory reporting of infectious diseases and published in Epidemiol kom vjesnik and Croatian Healthy—Stvenostatista	Retrospective cohort	National population	Law on the Common Action of Infectious Diseases Nar Nov 2007/79	1999 − 2008	2907	6.6
Portugal (de Carvalho and Núncio, 2006)	Centre for Vectors and Infectious Diseases Research Laboratory at the National Institute of Health, registry of nationally reported cases	Retrospective cohort	National population	Clinical with laboratory confirmation	1990 − 2004	628	0.4

Incidence estimates and 95% CIs have been rounded to the first decimal to the right for consistent level of precision.

CI, confidence interval; LB, Lyme borreliosis; PPY, population per year.

**Table 6.** Estimates of National Incidence of Lyme Borreliosis (Cases per 100,000 Population per Year) in Western Europe from the Literature Published from 2005 to 2020

Country	Data source	Study design	Study population	Case definition	Study period	No. of cases	Incidence (cases per 100,000 PPY) (95% CI)
Belgium (Vanthomme et al., 2012)	Belgian network of sentinel GPs	Prospective cohort	National population	EM—EUCALB	2003 − 2004		83.2 (73.6 − 92.7)
					2008 − 2009		90.2 (80.8 − 100.3)
Belgium (Geebelen et al., 2019)	Belgian network of sentinel GPs	Retrospective observational	1.3% of the population	EM—EUCALB	2015 − 2017	420	97.6 (82 − 113)
					2015		98 (81.8 − 114.2)
					2016		106.1 (90.1 − 122.2)
					2017		88.5 (74.3 − 102.8)
France (Letrilliart et al., 2005)	French Sentinels Network: 1178 sentinel GPs	Prospective cohort	National population	CDC, EUCALB	1999 − 2000	86 (LB), 77 (EM), 9 (LNB), 5 (LA)	9.4 (7.4 − 11.4)
France (Gueorguiev Penev et al., 2010)	France national hospitalization registry	Retrospective cohort, retrospective	Sixty-nine patients with ICD-10 codes for LB	LB—EUCALB	1999 − 2006	47 (LB), 5 (EM), 32 (LNB), 4 (LA), 3 (ACA), 2 (LC), 2 (ocular)	0.9 (0.8 urban, 1.1 rural)
Germany (Fulop and Poggensee, 2008)	Notifications to the RKI	Cross-sectional, retrospective, surveillance	Population of six regions where LB is notifiable	EM or LNB confirmed by analysis of CSF	2002–2006	23,394 (LB), 20,787 (EM), 799 (LNB)	
					2002		17.8
					2003		23
					2004		25
					2005		32
					2006		37.3
Germany (Adlhoch and Poggensee, 2010)	Notifications to the RKI	Retrospective cohort surveillance	Population of six regions where LB is notifiable	EM and LNB for 2002	2007 − 2009	16,461	37.8
					2002	3021	17.8
					2003	3977	23.5
					2004	4477	26.6
					2005	5461	32.6
					2006	6241	37.5
					2007	5680	34.3
					2008	5568	33.8
					2009	5213	31.7
Germany (Mehnert and Krause, 2005)	Notifications to the RKI	Retrospective	Population of six regions where LB is notifiable		2002	3019 (LB), 2697 (EM), 97 (LNB)	17.8
					2003	3968 (LB), 3442 (EM), 97 (LNB)	23.3
Germany (Enkelmann et al., 2018)	Notifications to the RKI	Retrospective cohort, surveillance	Population of nine regions where LB is notifiable	Clinical	2013 − 2017	56,446 (EM only), 53,177 (LB), 1481 (LNB), 1182 (LA)	33
					2013		41 (40.2 − 41.6)
					2015		26 (25.6 − 26.7)
The Netherlands (Hofhuis et al., 2015)	GP-based questionnaire and medical records review	Retrospective cohort	Forty-six percent of the population	LC	2009 − 2010	25^a^ 6^b^	0.18–0.34^a^ 0.04–0.08^b^
The Netherlands (Hofhuis et al., 2016)	Postal questionnaire to all GPs	Cross sectional	Sixty-two percent of the population	EM^c^	1994	4203^d^	38.6 (37.2 − 40.0)
					2001		74.3 (72 − 76.6)
					2005		103.8 (101.1 − 106.6)
					2009		133.9 (130.5 − 137.5)
					2014		139.6 (135.3 − 144.1)
Switzerland (Altpeter et al., 2013)	Mandatory surveillance database of tick-borne encephalitis, Federal Office of Statistics, data pool of santésuisse	Prospective cohort	National population	EUCALB definition	2008 − 2011	864	131 (124 − 142)
					2008	281	156
					2009	174	122
					2010	128	91
					2011	281	156

Incidence estimates and 95% CIs have been rounded to the first decimal to the right for consistent level of precision.

^a^ Crude incidence.

^b^ Adjusted incidence of LC.

^c^ GPs were sent surveys regarding the number of consultations of tick bites and diagnose of EM.

^d^ Number of GPs who responded to a postal questionnaire.

ACA, acrodermatitis chronica atrophicans; CDC, United States Centers for Disease Control and Prevention; CI, confidence interval; CSF, cerebrospinal fluid; EUCALB, European Union Concerted Action on Lyme Borreliosis; GP, general practitioner; ICD-10, International Classification of Diseases version 10; LA, Lyme arthritis; LB, Lyme borreliosis; LC, Lyme carditis; LNB, Lyme neuroborreliosis; PPY, population per year; RKI, Robert Koch Institute.

The review yielded 131 articles with either LB incidence estimates (*n* = 61) and/or reported number of LB cases (*n* = 70). Two countries (Bulgaria and Hungary) reported the number of LB cases but not the incidence. The articles that only reported numbers of LB cases are summarized in Supplementary Tables S7–S11 and are intended to provide additional insights.

### Study design

Study designs applied, case definitions utilized, and populations sampled varied markedly among the articles reviewed. Of the 61 articles with LB incidence estimates, 47 studies were retrospective, whereas 13 were prospective (Tables 2 − 6 and Supplementary Tables S1−S6). Among the retrospective studies, most (43 of the 47 studies) used a cohort study design, 3 were observational, and 1 was an ecological study. Most of the 61 studies used population-based data, but some studies enrolled defined subpopulations, such as inpatients and outpatients presenting with manifestations of LB, persons with facial palsy, or persons consulting general practitioners (GPs). The types of the studies published also varied, and thus, there may be risk of bias in the reported LB incidence estimates. In brief, the considerable heterogeneity makes data interpretation and comparability more complex.

All studies that met the inclusion criteria were retained and were not excluded based on study design, sample size, populations sampled, and so on. Studies are summarized by key variables to allow for interpretation of incidence estimates and potential biases or limitations.

### Use of case definitions published by European institutions

Case definitions used by the reviewed studies varied substantially, as shown in Tables 2–6. EUCALB definitions were used in 26 (20%) of the 131 articles presented in this report (13 that reported LB incidence and 13 that reported numbers of LB cases), of which 9 were from France. Other LB definitions in the published literature included clinical cases (*i.e*., consultations for tick bites or EM, cases of LC), laboratory-confirmed cases (*i.e*., LNB confirmed by analysis of cerebrospinal fluid), or cases of LB identified from databases using the International Classification of Diseases (ICD) or Read codes. The ECDC case definition for LNB (European Centre for Disease Prevention and Control, 2018), made reportable in 2018, was not employed in any studies.

### Incidence of LB in Europe

#### Incidence of LB in Eastern Europe region (Belarus, Czech Republic, Poland, Romania, Russia, Slovakia)

Five articles reported national incidence estimates of LB in Eastern European countries (Table 2). National incidence estimates for LB in Eastern European countries, considering any case definition, ranged from 0.9 to 46.8 cases per 100,000 population per year (PPY; Table 2). The highest LB incidence was in the Czech Republic (37.3/100,000 PPY between 2007 and 2016) (Kriz et al., 2018). The LB incidence in Poland was >20/100,000 PPY in 2007 and 2008 but was low (≤10/100,000 PPY) before 2004 (Stefanoff et al., 2014). Low incidences (<10/100,000 PPY) were reported in Belarus, Russia, and Slovakia (Dedkov et al., 2017, Karaban et al., 2009, Svihrova et al., 2011).

##### Subnational variation

Eleven articles estimated LB incidence at the subnational level in Eastern European countries reporting substantial variation (Supplementary Table S1). For example, after 2004, the incidence of LB in northern Slovakia ranged from 18.1 to 42.5/100,000 PPY, which is higher than the reported national LB incidence of 10.6 to 15.7/100,000 PPY (Bochničková et al., 2012). In Poland, LB incidence was highest in the eastern and northeastern regions. Within Podlaskie region, incidence was highest in the Sejny (215.1/100,000 PPY), Hajnówce (200.9/100,000 PPY), and Bielski (198.8/100,000 PPY) and lowest in the Zambrowski (29.8/100,000 PPY) and Łomżyński (38.5/100,000 PPY) counties (Supplementary Table S1) (Krzyżak et al., 2019).

#### Incidence of LB in Northern Europe region: Baltic States (Lithuania)

The only Baltic country with published LB incidence estimates was Lithuania (Supplementary Table S2), with data reported in only one article and at the subnational level. This study in the Vilnius district reported an incidence of LB of 85.4/100,000 PPY in outpatients attending an ambulatory unit in 2014–2016. LB cases were defined on the basis of documented clinical characteristics, laboratory results, electrocardiograms, and skin biopsy findings (Petrulioniene et al., 2021).

#### Incidence of LB in Northern Europe: Nordic region (Denmark, Finland, Norway, Sweden)

Four articles reported national incidence estimates of LB in Denmark, Finland, and Sweden (Table 3). National incidence estimates for LB in countries in Nordic region, considering any case definition, ranged from 1.9 to 7.3/100,000 PPY in studies reporting cases of LNB to 118/100,000 PPY in a study of clinically and microbiologically confirmed cases of LB (Table 3). Two articles reported incidences of LNB in Denmark, which ranged from 1.9 to 3.3/100,000 PPY between 1996 and 2015 (Dessau et al., 2015, Tetens et al., 2020). The highest LB incidence in Nordic region (118/100,000 PPY) was reported in Finland, which conducts national surveillance for clinically diagnosed LB cases and laboratory-confirmed LB cases (Sajanti et al., 2017). One study in Sweden evaluated different national data sources, and incidence of LNB was 6.2/100,000 population in 2014, with a higher incidence in the south (Dahl et al., 2019).

##### Subnational variation

An additional 13 articles estimated LB incidence at the subnational level in Denmark, Finland, Norway, and Sweden (Supplementary Table S3). For example, at the subnational level, the incidence of LNB in Denmark was higher in outlying islands (>10/100,000 PPY) and in southern Denmark (5.1/100,000 PPY) than in other regions (Dessau et al., 2015). In Finland, the highest LB incidence at the subnational level was observed in the Åland Islands, where incidence of clinically diagnosed EM was 884.6/100,000 PPY and laboratory-confirmed LB was 1597/100,000 PPY (Sajanti et al., 2017). The incidence of clinically diagnosed EM in other regions in Finland ranged from <5/100,000 PPY (Northern Ostrobothniam, Kainuu, and Lapland) to 161.8/100,000 PPY in South Karelia. The incidence of laboratory-confirmed LB followed a similar trend (Sajanti et al., 2017).

#### Incidence of LB in Northern Europe region: United Kingdom (England, Northern Ireland, Scotland, Wales) and Ireland

Six articles reported national incidence estimates of LB in countries in the United Kingdom and Ireland (Table 4), and five articles reported subnational LB incidences (Supplementary Table S4). National incidence estimates of LB in the United Kingdom considering any definition were <10/100,000 PPY, except for one study in Scotland, where reported LB incidence was 37.3/100,000 PPY (Table 4) (Cairns et al., 2019).

Published LB incidence estimates for the United Kingdom have used a variety of data sources and include insights into incidence at the wider UK level, at individual country levels, and at subnational regional levels within countries. Within the published literature, there was evidence of an impact of case definition on reported LB incidence. For example, a study in the GP-based Clinical Practice Research Datalink (CPRD; 2001 − 2012) found incidence of suspected and possible LB of 12.1/100,000 PPY for the United Kingdom and 37.3/100,000 PPY for Scotland (Cairns et al., 2019), whereas lower incidences were observed over a similar period (1998 − 2016) in another study using a primary case database with diagnosis of LB, suspected LB, or related conditions based on Read codes (Tulloch et al., 2020). Both studies showed an increase in LB incidence over time.

##### Subnational variation

There were an additional five articles that reported subnational LB incidence estimates in specific localities in the United Kingdom or Ireland (Supplementary Table S4). CPRD data showed a higher incidence of (suspected and possible) LB in southwest England (23.4/100,000 PPY) and lower incidence in northern England and West Midlands (6.3/100,000 PPY) (Cairns et al., 2019). National LB testing laboratory in Scotland reported an incidence of laboratory-confirmed LB of 6.8/100,000 PPY between 2008 and 2013; highest incidence was observed in the Highlands (44.1/100,000 PPY) (Mavin et al., 2015).

#### Incidence of LB in Southern Europe region (Croatia, Italy, Portugal, Spain)

Two articles reported national incidence estimates of LB for Portugal and Croatia (Table 5). The estimated national incidence of laboratory-confirmed LB (excluding EM) in Portugal was 0.4/100,000 PPY (de Carvalho and Núncio, 2006). The estimated national incidence of clinically diagnosed LB cases and laboratory-confirmed LB cases in Croatia was 6.6/100,000 PPY (Mulić et al., 2011).

##### Subnational variation

Five articles estimated LB incidence at the subnational level in specific localities in Croatia and Spain (Supplementary Table S5). In Croatia, for example, the highest LB incidence was reported in the northernmost provinces, ranging from 18.8 to 25.4/100,000 PPY (Mulić et al., 2011). In a study among confirmed positive Western plots and patients diagnosed with possible LB in Spain, the annual incidence of LB increased over the study period from 2.6 to 11.6/100,000 PPY (Vazquez-Lopez et al., 2015). In Lombardy (northern Italy), where there is mandatory reporting of LB to the Rare Disease Registry using diagnostic codes, incidence was 0.1/100,000 PPY between 2000 and 2015 (Zanzani et al., 2019).

#### Incidence of LB in Western Europe region (Belgium, France, Germany, Netherlands, Switzerland)

Eleven articles reported national incidence estimates of LB in Western European countries (Table 6). The national incidence of LB in countries in Western Europe ranged from 0.06 to 156/100,000 PPY. The highest LB incidence was observed in Switzerland using EUCALB definitions (156/100,000 PPY) (Altpeter et al., 2013). High LB incidences were also observed in Belgium and the Netherlands in studies that reported cases of EM or cases of EM and/or consultations for tick bites (106.1/100,000 PPY and 139.6/100,000 PPY, respectively) (Geebelen et al., 2019, Hofhuis et al., 2016).

The LB incidences were <10/100,000 PPY in France and 17.8–41/100,000 PPY in Germany (Table 6). In the Netherlands, periodic national postal surveys were sent to GPs to ascertain the incidence of consultations for tick bites and EM between 1994 and 2014 (Hofhuis et al., 2016), which found an increase in LB incidence from 38.6/100,000 PPY in 1994 to 139.6/100,000 PPY in 2014.

##### Subnational variation

There were 15 articles that reported LB incidence at the subnational level in specific localities in Western European countries (Supplementary Table S6), including France and Germany, which had the lowest national LB incidences. Incidence of EM in Belgium ranged from 30.9/100,000 PPY in East Flanders to 390.9/100,000 PPY in Limburg (Geebelen et al., 2019). Incidence of LB cases diagnosed according to the EUCALB definition as reported from the French Sentinel GP network was 180/100,000 PPY in Alsace and ranged from 30 to 511/100,000 PPY (Schmitt et al., 2006). LB incidence in Germany also showed substantial subnational variation: 0.3/100,000 PPY (Thuringa) to 90.1/100,000 PPY (Brandenburg) (Adlhoch and Poggensee, 2010).

#### Incidence of LB across all four regions of Europe

The highest LB incidences (>100 cases per 100,000 PPY) were reported in Belgium, Finland, the Netherlands, and Switzerland. Incidences were 20 to 40/100,000 PPY in the Czech Republic, Germany, Poland, and Scotland and <20/100,000 PPY in Belarus, Croatia, Denmark, France, Ireland, Portugal, Russia, Slovakia, Sweden, and the United Kingdom (England, Northern Ireland, and Wales). Higher incidence was observed at the subnational level than at the national level in eight countries, including the Republic of Ireland (up to 43 per 100,000 PPY), Scotland (up to 56.4 per 100,000 PPY), England (up to 23.4 per 100,000 PPY), Bulgaria (up to 30.9 per 100,000), Poland (up to 200.9 per 100,000 PPY), Russia (up to 40.5 per 100,000 PPY), Slovak Republic (up to 52.1 per 100,000 PPY), and Sweden (up to 464 per 100,000 PPY). Local studies conducted in Lithuania and Norway reported LB incidence of 85.4 and 552 per 100,000 PPY, respectively.

**FIG. 2.**
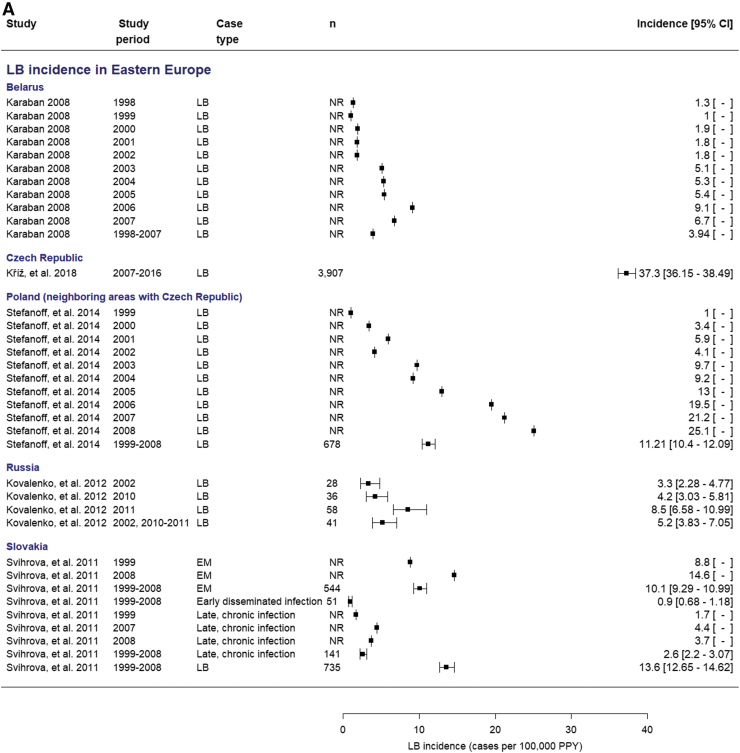

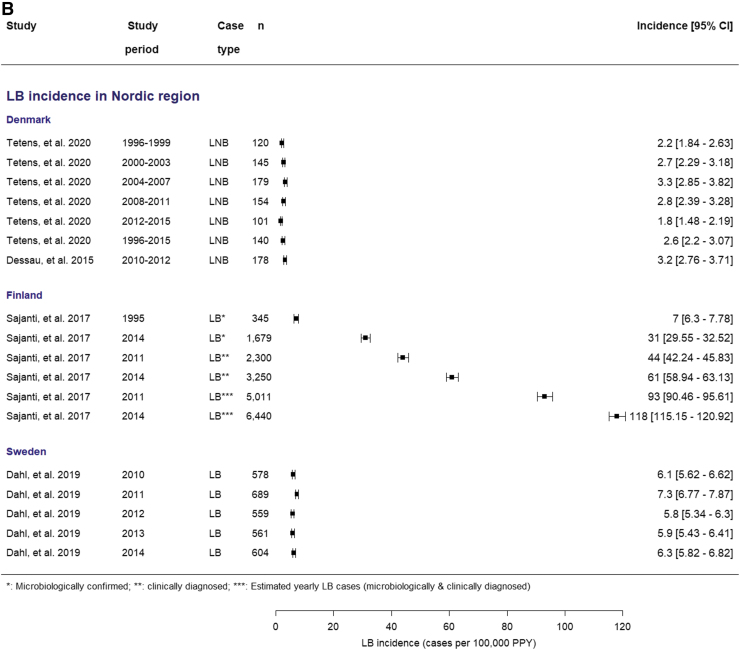

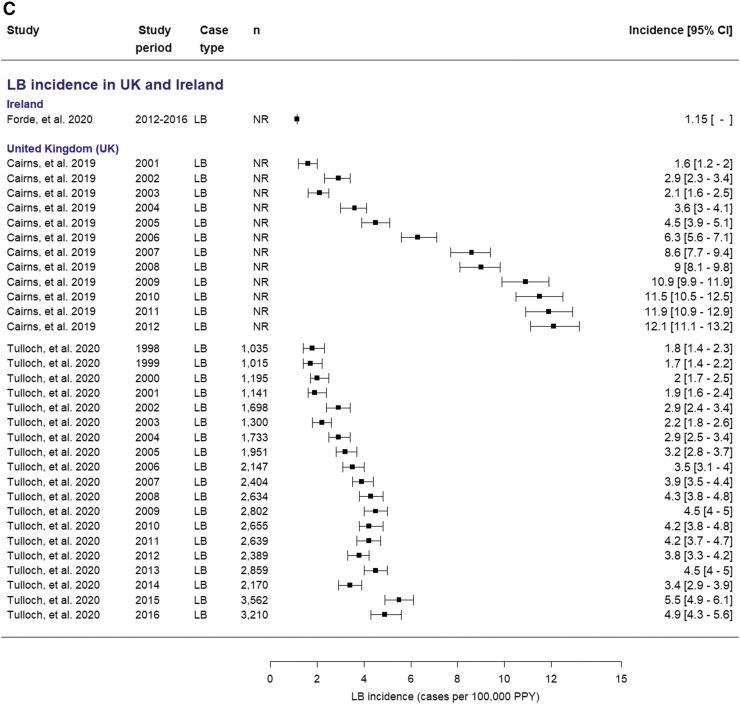

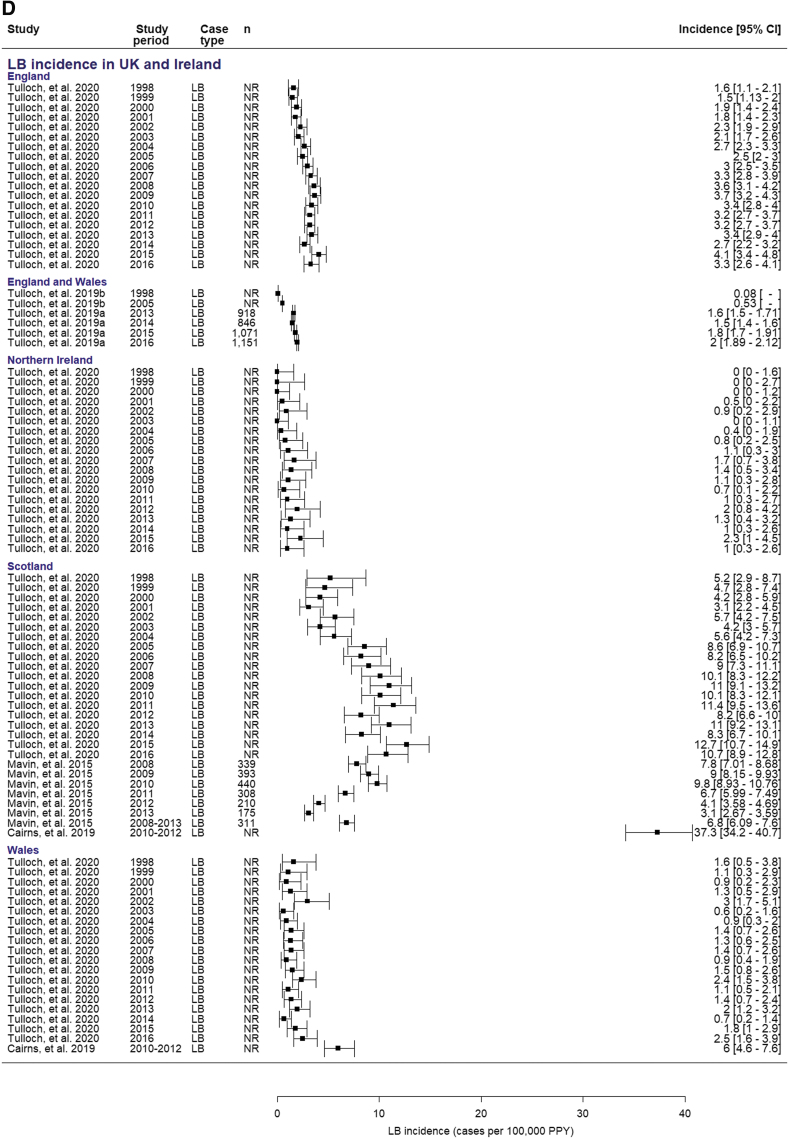

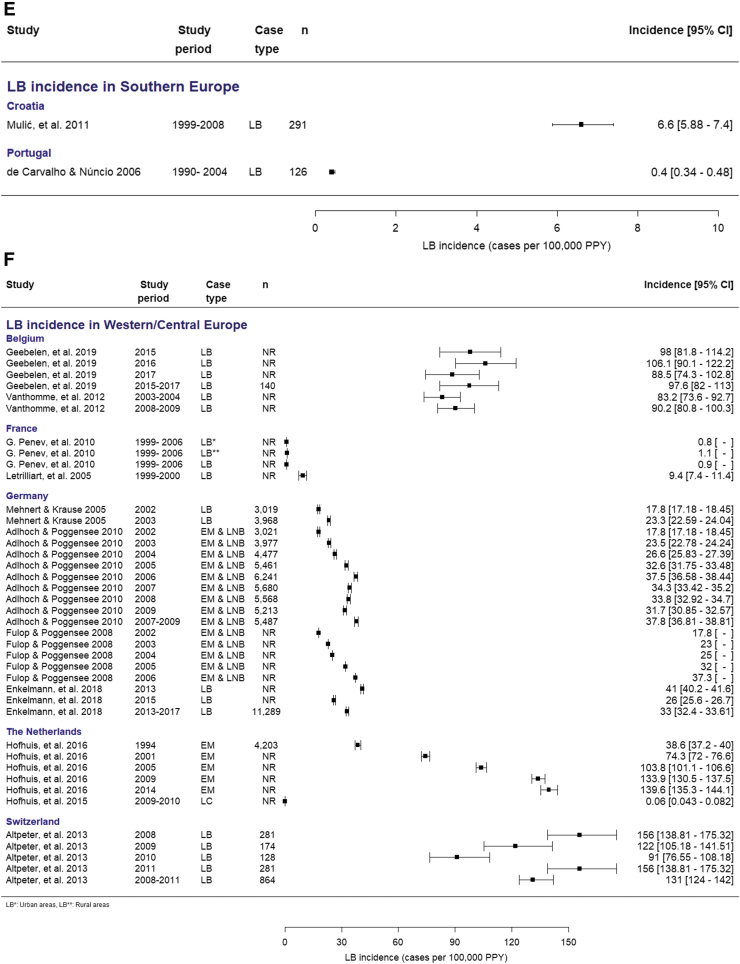
Forest plots of national-level annual incidence estimates in **(A)** Eastern Europe, **(B)** Northern Europe (Nordic region), **(C)** Northern Europe (The United Kingdom and Ireland), **(D)** Northern Europe (The United Kingdom and Ireland, England and Wales, Northern Ireland, Scotland, and Wales), **(E)** Southern Europe, and **(F)** Western Europe. Stratified by clinical manifestations—as applicable (when reported in study). EM, erythema migrans; LB, Lyme borreliosis; LC, Lyme carditis; LNB, Lyme neuroborreliosis; NR, not reported; PPY, population per year.

Forest plots of incidence estimates, with corresponding 95% CIs (when available from studies) are displayed in Fig. 2 to better visualize the data and compare across countries and European regions, and to view the varying and wide range of incidences and identify outliers.

Due to the considerable heterogeneity of the data (*I*^2^ > 80%) even after subanalyses by clinical manifestations and country–case definition interaction, we did not pool data and a meta-analysis was not reported (Campbell et al., 2020). Maps showing variation of LB incidence estimates across countries were also not developed due to the heterogeneity of the data. The high degree of heterogeneity indicates that incidence results should be interpreted in light of the complexity of data due to variability in study design, study setting, populations sampled, study periods, sample size, and case definitions utilized.

## Discussion

This review provides a unique and granular view of published estimates of LB incidence from epidemiological studies conducted in 25 countries in Europe from 2005 to 2020. Comparison of incidence across countries was limited by heterogeneity in study design and methods, including variations in the case definitions of LB. Four countries (Belgium, Finland, the Netherlands, and Switzerland) had national LB incidences >100/100,000 PPY, and three others (Czech Republic, Germany, and Poland) had national LB incidences of 20 to 40/100,000 PPY. National LB incidences were lower (<20/100,000 PPY) in Belarus, Croatia, Denmark, France, Ireland, Portugal, Russia, Slovakia, Sweden, and the United Kingdom (England, Northern Ireland, and Wales).

Furthermore, several countries had high LB incidences at the subnational level in some locales, indicating that national estimates of LB incidence may not accurately reflect the incidence at local levels. Heterogeneity in disease incidence between subnational localities could reflect true differences in disease risk. Alternatively, the differences could reflect the use of inconsistent epidemiological methods to assess LB incidence, highlighting the potential advantage of using standardized case definitions, such as those published by EUCALB.

Our systematic review complements a study that evaluated incidence as reported in national surveillance systems in European countries (Burn et al., 2023, in this issue). As noted in the surveillance study, among 25 countries where national surveillance incidence data were published, incidence data from the literature were also identified in 15 of these countries (Belgium, Croatia, Czech Republic, England, Finland, France, Germany, Ireland, Portugal, Russia, Poland, Scotland, Slovakia, Switzerland, and Wales). We found additional published estimates of LB incidence in the literature for six countries currently without national surveillance in place: Belarus, Denmark, Italy, the Netherlands, Spain, and Sweden. Studies conducted in these countries serve as important complementary data to inform the use of LB prevention strategies in the future.

Two systematic reviews have previously estimated LB incidence in Europe, both of which were limited to Western Europe. The systematic review by Sykes (2014) evaluated 11 studies from 18 Western European countries from database inception through 2013 and found a wide range of national LB incidences (0.0012 to 464/100,000 PPY) across the reported study years (1988 through 2011). The authors concluded that the LB incidence in Western Europe was 56.3/100,000 PPY, which equates to >200,000 cases per year (Sykes, 2014). A second systematic review across Western Europe by Vandekerckhove et al. (2021) evaluated 25 articles from 18 countries from database inception through 2018.

The authors found a similarly wide range of national LB incidence (0.001 to 632/100,000 PPY) across reported study years (1991 − 2017). Limited LB incidence data available from some countries in Western Europe were noted, and it was concluded that the incidence of LB was increasing in some countries, mainly in the northern and central regions (Vandekerckhove et al., 2021). In contrast to these previous articles, our review included 61 articles from 25 countries (including European countries outside of Western Europe) and provides a more comprehensive overview of the contemporary incidence of LB in Europe.

There are limitations in interpreting the epidemiological studies included in our review. Studies used different case definitions: clinical, clinical and/or laboratory confirmed, or laboratory confirmed. Sometimes different sources of data with different case definitions were even used within articles, such as Sajanti et al. (2017). Other times, different case definitions were used for studies from the same countries. This can be illustrated by the studies included for the United Kingdom. The study by Cairns et al. (2019) included all patients tested and treated for LB regardless of test results, which may have resulted in an overestimation of incidence. However, the study by Tulloch et al. (2019b) used only laboratory-confirmed cases and thus may have missed cases, leading to underestimation.

While the use of different case definitions limits directly comparing incidence estimates of LB, it provides important insights to the countries' LB disease burden. Many of the studies included in our review also captured various clinical manifestations of LB, further complicating matters. While the majority of cases reported were clinical cases of EM, there were also cases of clinical EM and/or laboratory confirmed, and less frequently, specific clinical manifestations, such as LNB, LA, or LC. Serology is the most frequently used method of laboratory confirmation, which is supported by the results of our review. Nonetheless, many patients who present with EM, which is the feature most commonly used to diagnose Lyme disease, will have negative antibody test results. Compounding potential confusion is the fact that in patients with no clinical evidence of the disease, who have a low probability of infection, antibody assays for LB are likely to yield false-positive results (Shapiro, 2014).

The differences in incidence estimates and in clinical manifestations across countries in Europe were expected due to varying distributions of *Bbsl* across Europe, influenced by a range of factors—including geographical, environmental, and climate factors, compounded by human recreational and occupational risk factors, and so on (Van den Wijngaard et al., 2017). We did not exclude nor restrict our review to specific case definitions; rather, the variability in case reporting across countries complements incidence estimates from national surveillance systems in our companion article (Burn et al., 2023, in this issue). Given the wide range of incidence estimates of LB, heterogeneity in how varying case definitions were used in these measures sheds valuable insights into the disease across the region. Nonetheless, factors contributing to heterogeneity in varying study designs, case definitions utilized, data sources, and diagnostic methods should be considered when interpreting our results.

Most studies in the review are epidemiological studies. We did not perform a quality assessment, formally rank them on basis of quality of evidence, and consequently exclude certain articles and other data sources. While we realize that systematic reviews are susceptible to varying quality of studies that arise in any of the included primary studies, we sought to obtain estimates of incidence across Europe from published epidemiological studies. In an effort to be comprehensive, we did not want to exclude data that could give important insight into regions that may have LB burden that is not captured routinely from surveillance systems.

Nevertheless, differences in quality of design and implementation of these epidemiological studies do exist. For example, in a study in our review conducted in Russia, the authors sought to evaluate effectiveness of areas treated with Baytex to eliminate ticks but not to specifically measure incidence (Bogachkina et al., 2011). The methodology is not clear, and the authors did not report case definition utilized, which are limitations in the interpretation of this incidence estimate for Russia; however, given this study met our inclusion criteria and reported incidence in their findings, we still report results here as part of our review.

Alternatively, a study conducted in Belgium among a representative sample of the national population clearly described their methodology, utilized EUCALB case definitions, and addressed possible information bias for EM (Vanthomme et al., 2012). Appropriate statistical analyses conducted to compare incidence rates using the Belgian population were clearly described, and the authors clearly stated the strengths and limitations of their study for full interpretation.

Our data may underestimate the future LB burden. The human health burden of LB is expected to increase in Europe as the range of tick populations expands (altitude and latitude), potentially as a result of the impact of climate change on tick life cycles, migratory animals, and human activities (Hussain et al., 2021). Furthermore, the LB incidence in countries in Europe is likely underestimated due to limitations of public health surveillance and epidemiological studies. For example, LB may be present in countries where data are currently absent, including at subnational levels.

New prevention methods for LB are on the horizon. A prophylactic monoclonal antibody for LB pre-exposure prophylaxis under development has demonstrated potential to offer protection (Schiller et al., 2021). Furthermore, a vaccine is currently in clinical development (ClinicalTrials.gov Identifier: NCT04801420). Recent studies have highlighted the value of a vaccine for the prevention of LB based on high acceptability and the limited capability of existing measures to prevent tick-borne diseases (Hook et al., 2021, Schwartz et al., 2022).

Data presented in this review, and in the LB incidence in Europe from the National Public Health Surveillance Systems (Burn et al., 2023, in this issue) companion article, indicate that the incidence of LB disease in Europe is substantial but geographically heterogeneous, both among and within countries. Data reported at the national level can often mask subnational differences, particularly in areas with substantially higher incidence. Incidence data from the epidemiological studies included here can help identify subnational regions of high incidence. Therefore, data from this review serve as an important complement to incidence data from national surveillance systems, where subnational data may not always be available or reported in certain regional areas. Collectively, these data can be used to identify countries and localities with a high LB disease burden that may benefit from future preventive and therapeutic strategies, including a vaccine, to optimize reduction in LB disease burden.

## Supplementary Material

Supplemental data
