# Supplementary material for: Incidence of Lyme Borreliosis in Europe: A Systematic Review (2005–2020)
Source: Vector Borne Zoonotic Dis. 2023 Apr 12;23(4):172–94. doi: 10.1089/vbz.2022.0070 (PMC10122234; doi:10.1089/vbz.2022.0070)
Supplement: Supplemental data [file Supp_TableS1-S11.pdf]

Incidence of Lyme borreliosis in Europe: a systematic literature review

SUPPLEMENT

Table S1: Incidence (cases per 100,000 PPY) of LB in sub-national areas of Eastern European countries from literature published from 2005 to 2020

Country (Ref)	Sub-national area	Author Year	Data source	Study Population	Cases (n)	Study design	Study period	Case definition	Incidence (95% CI)
Czech Republic [1]	Regional Bohemia	Daniel 2008	National surveillance data	Regional general population	7,391	Retrospective observational	1999-2004	NR	12.0 (11.4-12.7)
Poland [2]	Bialystok North-east Poland	Toczylowski 2020	Hospital records	Children <18 years	26	Retrospective cohort, observational	2015-2019	Clinical with laboratory confirmation	2.5 (1.1-6.0)
Poland [3]	Lubuskie	Zbrzezniak 2019	Mandatory surveillance system	National general population except Bialystok district	798	Retrospective observational, surveillance	2017	Surveillance definition	78.5
	Dolnoslaskie				852				29.4
	Kujawsko-Pomorskie				545				26.2
	Lubelskie				1975				92.8
	Malopolskie				3322				98.1
	Mazowieckie				2216				41.2
	Opolskie				644				65
	Podkarpackie				1480				69.6
	Podlaskie				1542				130.1
	Pomorskie				1466				63.2
	Slaskie				2779				61
	Swietokrzyskie				450				36
	Warminsko-mazurskie				1302				90.7
	Wielkopolskie				655				18.8
	Zachodniopomorskie				851				49.9
	Ladzkie				637				25.7
Poland [4]	Kujawsko-Pomorskie	Paradowska-Stankiewicz 2015	Mandatory surveillance system	National general population	430	Retrospective cross-sectional	2013	Clinical and laboratory criteria	20.5
	Lubelskie				816				37.8
	Lubuskie				350				34.2
	Malopolskie				1817				54.1
	Mazowieckie				1469				27.2
	Opolskie				431				42.8
	Podkarpackie				988				46.4
	Podlaskie				1199				100.2
	Pomorskie				507				22.1

	Wielkopolskie				241				7
	Zachodniopomorskie				445				25.9
	DolnoÅ›laskie				586				20.1
	LÃ³dzkie				291				11.6
	ÅšlÄskie				2278				49.5
	Åšwietokrzyskie				189				14.9
	WarmiÅsko-Mazurskie				736				50.8
Poland [5]	Podlaskie voivodship	Krzyzak 2019	Provincial Sanitary-Epidemiological Station in Bialystok. National bulletins	National general population	NR	Retrospective cross-sectional, surveillance	2011-2018	Clinical, laboratory, tick exposure	104.2
	Bialystok								88.5
	Sokolka								172.9
	Bielsk Podlaski								198.8
	Wysokie Mazowie-ckie								48.5
	AugustÃw								91.6
	ÅomÅa								38.5
	Grajewo								167
	Siemiatycze								111
	HajnÃwka								200.9
	ZambrÃw								29.8
	MoÅki								51.1
	Kolno								60
	SuwaÅki								154.5
	Sejny								215.1
Poland [7]	Lubuskie	Stefanoff 2006	Routine epidemiological surveillance + Sero-epidemiological studies	National general population (except Bialystok)	19,052	Retrospective surveillance, sero-epidemiological	1999-2005	Clinical, laboratory	
							1999		0.49
							2004		12.3
	Opolskie						1999		1.0
							2004		16.3
Romania [8]	Mures county, central-northern region	Tilea, 2014	120 consecutive inpatients	Regional general population	44 (LB) 28 (EM) 13 (LNB) 1 (LC	Prospective, sero-epidemiological	2010	CDC and EUCALB	7.47
					76 (LB) 49 (EM)		2011		12.91

					14 (LNB)<br>2 (LA)				
Russia [9]	St. Petersburg	Bogachkina 2011	Centre of Hygiene and Epidemiology, The Directorate of the Federal Service for Surveillance on Consumer Rights Protection and Human Well-Being	General population	487<br>307	Cross-sectional, Surveillance	2009<br>2010	NR	10.7<br>6.7
Slovak Republic [10]	RuĬomberok and Liptovský Mikuláš	Bochnickova 2012	Medical records	Patients attending Infectious Disease Departments	476	Retrospective cohort	1989-2010<br><br>1989<br>1990<br>1991<br>1992<br>1993<br>1994<br>1995<br>1996<br>1997<br>1998<br>1999<br>2000<br>2001<br>2002<br>2003<br>2004<br>2005<br>2006<br>2007<br>2008<br>2009<br>2010	Epidemiological history, clinical, laboratory findings	7.5<br><br>3.0<br>1.5<br>3<br>0.7<br>2.2<br>6.6<br>16.4<br>52.1<br>9.7<br>10.4<br>3.0<br>14.8<br>11.1<br>9.8<br>9,0<br>18.1<br>30.2<br>25.7<br>32.5<br>42.3<br>24,0<br>21.2

ACA, Acrodermatitis chronica atrophicans; CDC, the United States Centers for Disease Control and Prevention; ECG, electrocardiograph; EUCALB, European Union Concerted Action on Lyme borreliosis, EM erythema migrans; LA Lyme arthritis; LC, Lyme carditis; LNB Lyme neuroborreliosis

*Incidence ***proportion*** reported, rather than incidence *rate* (person-time).

Table S2: Incidence (cases per 100,000 PPY) of LB in sub-national areas of the Baltic States from literature published from 2005 to 2020

Country (Ref)	Sub-national area	Author Year	Data source	Study Population	Cases (n)	Study design	Study period	Case definition	Incidence (95% CI)
Lithuania [11]	Vilnius district	Petrulioniene, 2020	Clinical records, Center of infectious Diseases, Vilnuius	Outpatients	7424	Retrospective cohort, observational	2014-2016<br>2016	Clinical, laboratory, ECG, skin biopsy findings.	85.4<br>101.6

ACA, Acrodermatitis chronica atrophicans; LB Lyme borreliosis; EM erythema migrans; LA Lyme arthritis; LC, Lyme carditis; LNB Lyme neuroborreliosis.

*Incidence ***proportion*** reported, rather than incidence *rate* (person-time).

Table S3: Incidence (cases per 100,000 PPY) of LB in sub-national areas of Nordic region countries from literature published from 2005 to 2020

Country (Ref)	Sub-national area	Author Year	Data source	Study Population	Cases (n)	Study design	Study period	Case definition	Incidence (95% CI)
Denmark [12]	Zealand	Dessau 2015	Danish notification system for infectious diseases, Danish microbiology database, Danish civil registration system.	Population of Denmark	65	Prospective cohort	2010-2012	LNB	2.6
	Capital (Denmark)				115				2.3
	Southern Denmark				182				5.1
	Middle Jutland				129				3.4
	Northern Jutland				35				2.0
Denmark [13]	Funen	Andreasen 2020	Confirmed LNB cases and controls	10 municipalities of Funen	401	Case-control	1995-2014	LNB: Clinical, laboratory confirmation	
							1998		2.33
							2006		7.93
Denmark [14]	Islands of Funen and Langeland	Knudtzen 2017	University Hospital based data	LNB cases verified by positive Borrelia intrathecal antibody	431	Retrospective cohort	1995-2014	Positive CSF IgM and/or IgG	4.7
Denmark [15]	North Jutland County	Krabbe 2008	GP medical records	Patients with LNB North Jutland County	84	Retrospective cohort	1998-2006	Detection of intrathecal antibodies	2.0
Finland [16]	Ãland Islands	Sajanti 2017	3 Registers: Register for Primary Health Care Visits (Avohilmo); National Infectious Diseases Register (NIDR); National Hospital Discharge Register (Hilmo)	Finnish national general population	11,793 (Avohilmo)/ 21,051 (NIDR)	Retrospective cohort	2010-2014	GP diagnosed EM/disseminated laboratory conformed LB	884.6/1597.0
	Southwest Finland								83.3/46.3
	Helsinki and Uusimaa								63.1/34.4
	Satakunta								28.4/13.2
	Satakunta								17.3/3.9
	Pirkanmaa								17.1/1.5
	Päijät-Häme								29.9/10.8
	Kymenlaakso								142.9/56.7
	South Karelia								161.8/48.7
	Southern Savonia								47.0/13.3
	Eastern Savonia								81.0/14.7
	North Karelia								66.4/15.8
	Northern Savonia								47.5/14.2
	Central Finland								49.7/7.8
	Southern Ostrobothnia								18.2/1.8
	Vaasa								40.3/31.2
	Central Ostrobothnia								29.4/8.7
	Northern Ostrobothnia								4.8/1.3
	Kainuu								4.2/1.8
	Länsi-Pohja								10.8/3.1
	Lapland								5.5/3.9

Norway [17]	South-Rogaland,	Tveitnes 2012	Hospital medical and laboratory records	62,000 children <14 years of age	142	Retrospective cohor	2001-2009	Clinical, laboratory (LNB)	26
Norway [18]	Vestfold, Telemark, Aust-Agderand Vest-Agder	Eliassen 2017	GP medical records, surveillance data	4 counties with highest LD incidence	6,565	Retrospective cohort	2005-2009<br>2005<br>2006<br>2007<br>2008<br>2009<br>2005-2009<br>2005<br>2006<br>2007<br>2008<br>2009	LB<br><br><br><br><br><br>EM	19.70 (8.6-30.9)<br>22.1 (10.9-37.8)<br>22.6 (6.3-42)<br>21.4 (8.5-37.9)<br>18.9 (4.4-36)<br>13.4 (13.1-14.3)<br>448 (339-574)<br>353 (257-426)<br>552 (399-714)<br>450 (371-571)<br>455 (320-627)<br>431 (328-541)
Sweden [19]	South-East Sweden, Blekinge County	Bennet 2006	Electronic medical records	Primary healthcare clinics	3,437	Retrospective cohort	1997-2002<br>1997<br>1998<br>1999<br>2000<br>2001<br>2002	EM – CDC, EUCALB	464<br>364<br>400<br>264<br>664<br>464<br>632
Sweden [20]	Gothenburg or Ãcker, Kung, Tjrn, Stenungsund, Ale, Lerum, Partille,HÃryda, MÃlndal, Kungsbacka	Sodermark 2017	Medical records of children (< 15 years) treated for LNB	Gothenburg and regional hospitals	548	Retrospective cohort	2002-2014<br>2005<br>2010	Clinical	28<br>42<br>14

CDC, the United States Centers for Disease Control and Prevention; CI, confidence interval; CSF, cerebrospinal fluid; EM, erythema migrans; EUCALB, European Union Concerted Action on Lyme borreliosis; GP, general practices; LNB, Lyme neuroborreliosis; Avohilmo, Register for Primary Health Care Visits; NIDR, National Infectious Diseases Register

*Incidence ***proportion*** reported, rather than incidence *rate* (person-time).

Table S4: Incidence (cases per 100,000 PPY) of LB in sub-national areas of the UK and Ireland from literature published from 2005 to 2020

Country (Ref)	Sub-national area	Author Year	Data source	Study Population	Cases (n)	Study design	Study period	Case definition	Incidence (95% CI)
Ireland [21]	Connemara	Vellinga 2018	All LB tests University Hospitals Galway laboratory	2,314,374 requests over the 5-year period	242	Prospective cohort	2010-2014	EIA and LIA for IgG or IgM.	43
	Loughrea								12
	County Roscommon								11
	West of Ireland overall								2
Scotland [22]	Ayrshire & Arran	Mavin 2015	National Lyme Borreliosis Testing laboratory and questionnaires from all laboratory confirmed cases within NHS Highland	Scotland national general population	42	Retrospective cohort, sero-epidemiological	2008-2013	ELISA (IgM/IgG), then immunoblot	1.9
	Tayside				223				9.2
	Dumfries & Galloway				56				6.3
	Lothian				163				3.3
	Fife				45				2.1
	Greater Glasgow & Clyde				320				4.5
	Highlands				831				44.1
	Lanarkshire				59				1.7
	Borders				21				3.1
	Western Isles				22				13.8
	Forth Valley				26				NR
	Grampian				57				NR
Scotland [23]	Highlands	Milner 2009	Surveillance data National Lyme Borreliosis Testing Laboratory at Raigmore Hospital in Inverness.	Scotland population	NR	Cohort, Sero-epidemiological, Surveillance	2007-2008	LB as assessed by serology/Western blot	43.4
Scotland [24]	Highlands	Slack 2011	National LB testing Laboratory, Medical microbiology Department at Ninewells Hospital & Medical School, Dundee	Samples from Tayside and the Highlands April 2001-March 2010	78	Retrospective Longitudinal	2006-2007	Weak positive or positive Western blot	25.4
					175		2009-2010		56.4
	Tayside				10		2001-2002		2.57
					67		2009-2010		16.8
	Rest of Scotland				-		2005-2006		0.8
					-		2006-2007		0.8
							2009-2010		5.5
United Kingdom [25]	South West	Cairns 2019	CPRD	8% of the population	248	Retrospective	2010-2012	Read codes for LB, suspected and possible LB	23.4 (20.6-26.6)
	London				117				7.7 (6.3-9.3)
	South				425				13.9 (12.9-15.3)
	East England				108				8.1 (6.6-9.8)
	West Midlands				70				6.3 (4.9-8.0)
	North				162				6.3 (5.3-7.3)

CI, confidence interval; CRPD, Clinical Practice Research Database; ELISA, enzyme linked immunosorbent assay; LB, Lyme borreliosis; LIA, line immunoassay; NR, not reported.

*Incidence ***proportion*** reported, rather than incidence *rate* (person-time).

Table S5: Incidence (cases per 100,000 PPY) of LB in sub-national areas of Southern European countries from literature published from 2005 to 2020

Country (Ref)	Sub-national area	Author Year	Data source	Study Population	Cases (n)	Study design	Study period	Case definition	Incidence
Republic of Croatia [26]	Sibensko-kninska	Mulic 2011	Mandatory reporting to Croatian Institute for Public Health and Health	National general population	0	Retrospective cohort	1999-2008	NR	0
	Osjecko-Baranjska				71				2.1
	Viroviticko podravska				57				6.1
	Istarska				58				2.8
	Vukovarsko-Srijemska				35				1.7
	Krapina-Zagorje				362				25.4
	Zadarska				2				0.1
	Zagreb				301				9.7
	City of Zagreb				992				12.7
	Bjelovarsko-Bilogoraska				56				4.2
	Koprivnicko-krizevacka				255				20.5
	Primorsko-goranska				191				6.3
	Pozesko-Slavonska				35				4.1
	Licko-senjska				3				0.6
	Karlovacka				67				4.7
	Sisacko-moslavacka				65				3.5
	Spliskot-Dalmatinska				13				0.3
	Dubrovacko-neretvanska				6				0.5
	Medimurska				223				18.8
	Varazdinska				68				3.7
	Brodsko-posavska				47				2.7
Republic of Croatia [27]	Bjelovarsko-bilogorska county	Ropac 2013	Institute of Public Health, Bjelovar-Bilogora	3% of the national general population	32	Retrospective	2007-2011	EM	4.5
Italy [28]	Lombardy	Zanzani 2019	The Rare Disease Register of the Lombardy Region.	National general population	189	Prospective observational	2000-2015	Codes and Read codes specific to Lyme disease	0.124
							2005		0.03
							2014		0.26
	Lodi province						2000-2015		0.03
	Sondrio province						2000-2015		0.8
Spain [29,30]	Lugo Province	Vazquez-Lopez 2015	Medical records	Persons with a positive Western Blot, inpatients with possible LB	108 (LB)	Retrospective cohort	2007	CDC	2.6
							2012		11.6
Spain [31]	Lugo Province	Vazquez-Lopez 2016	Hospital medical records	Children (<15 years)	10	Retrospective cohort	2006-2013	CDC	5.5

CDC, the United States Centers for Disease Control and Prevention; EM, erythema migrans; EUCALB, European Union Concerted Action on Lyme borreliosis; LA, Lyme arthritis; LB, Lyme borreliosis; NR, not reported.

*Incidence ***proportion*** reported, rather than incidence *rate* (person-time).

Table S6: Incidence (cases per 100,000 PPY) of LB in sub-national areas of Western/Central European countries from literature published from 2005 to 2020

Country (Ref)	Sub-national area	Author Year	Data source	Study Population	Cases (n)	Study design	Study period	Case definition	Incidence (95% CI)
Belgium [32]	East Flanders<br>Limburg<br>Flemish region<br>Walloon region<br>Brussels Capital	Geebelen 2019	Belgian network of sentinel GPs	National general population (inhabitants, 1.3% of the population)	420	Retrospective observational	2015-2017	EM: EUCALB	30.9 (21.4-40.3)<br>390.9 (329.7-451.9)<br>126 (101.0-150.9)<br>74.2 (68.3-80.1)<br>34.0 (28.4-39.6)
France [33]	Alsace	Raguet 2018	GP surveillance (388 GPs)	National general population	672 (LB)<br>530 (EM)<br>68 (LA)<br>54 (LNB)<br>6 (ACA)<br>1 (LC)<br>1 (ophthalmic)	Retrospective cohort	2014-20152014<br>2015	EUCALB	117(109-126)<br>121 (109-133)<br>113 (101-125)
France [34]	Limousin<br>Alsace<br>Pays de la Loire<br>Provence-Alpes-Côtes-d'Azur	Septfons 2019	National Sentinel GP network (2011-2016), national hospital discharge database (2005-2016)	National general population	667	Retrospective observational	2011-2016	Clinical with laboratory confirmation	239 (68-410)<br>148 (45-251)<br>5 (0-27)<br>8 (0-20)
France [35]	Meuse	Beytout 2007	Physician reports to the National Reference Center	Residents of 2 rural regions	25 (LB)<br>16 (EM)<br>1 (LA)	Prospective cohort	2003	EUCALB	84 (47-121)
					49 (LB)<br>37 (EM)<br>1 (LA)<br>1 (LC)		2004		156 (85-227)
					35 (LB)<br>28 (EM)		2005		106 (55-157)
	Puy-de-Dôme				90 (LB)<br>51 (EM)<br>1 (LA)<br>1 (LC)<br>1 (LNB)		2004		117 (76-159)
					62 (LB)<br>33 EM)<br>4 (LA)<br>1 (LNB)		2005		76 (38-114)
France	Franche-Comté	Tessier 2018			432 (LB)		2010-2012	EUCALB	85 (77-92)

Country (Ref)	Sub-national area	Author Year	Data source	Study Population	Cases (n)	Study design	Study period	Case definition	Incidence (95% CI)
[36]			GP-based sentinel surveillance	Regional population attending GPs	392 (EM)<br>4 (LA)<br>31 (LNB)<br>3 (Lymphocytoma)<br>1 (ACA)	Prospective cohort			
	Doubs								73
	Haute-Saone								77
	Jura								100
	Territoire de Belfort								113
France [37]	Alsace	Schmitt 2006	GP-based sentinel surveillance	Regional population attending GPs	1,365 (LB)<br>891 (EM)<br>291 (LA)<br>254 (LNB)<br>29 (Cutaneous-secondary or tertiary)<br>3 (LC)	Prospective cohort	2001-2003	EUCALB	180<br>(30-511 by canton)
	Limousin				217 (LB)<br>170 (EM)				NR
	National	Letrilliart 2005	French Sentinels Network: 1178 sentinel GPs	National general population	86	Prospective cohort	1999 2000	CDC, EUCALB	9.4 (7.4-11.4)
France [38]	Provence–Alpes–Côte d'Azur								0 (0-7)
	Alsace								86 (51-134)
Germany [39]		Fulop 2008	Notifications to the Robert Koch Institute	Population of 6 regions where LB is notifiable	23,394	Cross-sectional; Retrospective surveillance	2002-2006	EUCALB	
	Brandenburg						2006		77.6
	Berlin						2006		5.7
Germany [40]	Bavaria	Heinzinger 2017	Sentinel Lyme Disease Incidence Study initiated in mid-2012, routine surveillance	Regional general population	16,439 (LB)<br>15,797 (EM)<br>373 (LA)<br>302 (LNB)	Retrospective cohort, surveillance	2013-2016	EM: clinical only, LNB, LA: clinical and laboratory	34

Country (Ref)	Sub-national area	Author Year	Data source	Study Population	Cases (n)	Study design	Study period	Case definition	Incidence (95% CI)
Germany [41]	6 states	Mehnert 2005	Notifications to the Robert Koch Institute	Population of 6 regions where LB is notifiable	3019	Retrospective	2002	Clinical and/or lab confirmed	
					3968		2003		
	Berlin						2002		2.0
							2003		3.0
	Brandenburg						2002		56.7
							2003		71.7
	Meckl. Vorpommern						2002		7.4
							2003		8.7
	Sachsen						2002		23.8
							2003		29.6
	Sachsen Anhalt						2002		12.3
							2003		12.9
	Thuringen						2002		0.3
							2003		10.2
Germany [42]	All regions	Adlhoch 2010	Notifications to the Robert Koch Institute	Population of 6 regions where LB is notifiable	16,461 (LB)	Retrospective cohort, surveillance	2007-2009		-
	Berlin				69		2002	LB, EM, LNB	2.0
					103		2003	LB, EM, LNB	3.0
					163		2004	LB, EM, LNB	4.8
					226		2005	LB, EM, LNB	6.7
					173		2006	LB, EM, LNB	5.1
					137		2007	LB, EM, LNB	4.0
					103		2008	LB, EM, LNB	3.0
					79		2009	LB, LA, EM, LNB	2.3
	Brandenburg				1,467		2002	LB, EM, LNB	56.8
					1,860		2003	LB, EM, LNB	72.2
					2,024		2004	LB, EM, LNB	78.8
					2,306		2005	LB, EM, LNB	90.1
					2,193		2006	LB, EM, LNB	86.1
					2,048		2007	LB, EM, LNB	80.8
					1,787		2008	LB, EM, LNB	70.8
					1,797		2009	LB, LA, EM, LNB	71.2
	Mecklenburg-Western Pomerania				129		2002	LB, EM, LNB	7.4
					151		2003	LB, EM, LNB	8.7
					180		2004	LB, EM, LNB	10.5
					374		2005	LB, EM, LNB	21.9
					453		2006	LB, EM, LNB	26.7
					529		2007	LB, EM, LNB	31.5
					650		2008	LB, EM, LNB	39.1

Country (Ref)	Sub-national area	Author Year	Data source	Study Population	Cases (n)	Study design	Study period	Case definition	Incidence (95% CI)
					730		2009	LB, LA, EM,<br>LNB	43.9
	Saxony				1,034		2002	LB, EM, LNB	23.8
					1,289		2003	LB, EM, LNB	29.8
					1,464		2004	LB, EM, LNB	34.1
					1,636		2005	LB, EM, LNB	38.3
					2,216		2006	LB, EM, LNB	52.1
					1,937		2007	LB, EM, LNB	45.9
					1,911		2008	LB, EM, LNB	45.6
					1,668		2009	LB, LA, EM,<br>LNB	39.8
	Saxony-Anhalt				314		2002	LB, EM, LNB	12.3
					329		2003	LB, EM, LNB	13
					381		2004	LB, EM, LNB	15.3
					477		2005	LB, EM, LNB	19.3
					531		2006	LB, EM, LNB	21.7
					604		2007	LB, EM, LNB	25
					576		2008	LB, EM, LNB	24.2
					331		2009	LB, LA, EM,<br>LNB	13.9
	Thuringia				8		2002	LB, EM, LNB	0.3
					245		2003	LB, EM, LNB	10.3
					265		2004	LB, EM, LNB	11.3
					442		2005	LB, EM, LNB	18.9
					675		2006	LB, EM, LNB	29.2
					425		2007	LB, EM, LNB	18.6
					541		2008	LB, EM, LNB	23.9
					608		2009	LB, LA, EM,<br>LNB	26.8
Germany<br>[116]	East Germany	Wilking<br>2014	Notifications to the Robert Koch Institute	Retrospective surveillance	18,894 (LB)<br>18,016 (EM)<br>367 (LA)<br>630 (LNB)		2009-2012		34.9
							2009		
							2012		19.54
	Mecklenburg-Western Pomerania						2011		74.8
	Berlin						2012		1.2
Netherlands<br>[43]	Apeldoorn and Zutphen	Bierman<br>2019	Hospital records of patients with facial palsy	Adults with facial palsy visiting the departments of	26	Retrospective cohort	2007-2017	LNB<br>Clinical and laboratory	0.9 (0.6-1.3)

Country (Ref)	Sub-national area	Author Year	Data source	Study Population	Cases (n)	Study design	Study period	Case definition	Incidence (95% CI)
				neurology and/or otorhinolaryngology of Gelre hospitals					
Netherlands [44]	Amsterdam	Botman 2018	Medical records	Medical records of 56,996 patients registered in 12 GPs	2,619	Observational	2010- 2015	Immunoblot confirmed cases presenting to GPs	880
Switzerland [45]	*All regions*	Altpeter 2013	National surveillance database	National general population	864	Prospective cohort	2008-2011	EUCALB	
	Region 1: Geneva, Neuchâtel, Vaud, Valais						2008-2011		67
							2008		59
							2009		60
							2010		55
							2011		93
	Region 2: Berne, Fribourg, Jura						2008-2011		154
							2008		184
							2009		113
							2010		123
							2011		197
	Region 3: Aargau, Basel, Solothurn						2008-2011		130
							2008		161
							2009		170
							2010		86
							2011		105
	Region 4: Lucerne, Obwalden, Nidwalden, Schwyz, Uri, Zug						2008-2011		158
							2008		154
							2009		102
							2010		127
							2011		248
	Region 5: Appenzell, Glarus, St. Gallen, Schaffhausen, Thurgau, Zurich, Lichtenstein						2008-2011		156
							2008		202
							2009		151
							2010		88
							2011		185
	Region 6: Grisons, Ticino						2008-2011		128
							2008		168
							2009		124
							2010		96
							2011		125

ACA, Acrodermatitis chronica atrophicans; CDC, the United States Centers for Disease Control and Prevention; CI, confidence interval; EM, erythema migrans; EUCALB, European Union Concerted Action on Lyme borreliosis; LA, Lyme arthritis; LB, Lyme borreliosis; LC, Lyme carditis; LNB, Lyme neuroborreliosis; NR, not reported

*Incidence ***proportion*** reported, rather than incidence *rate* (person-time).

Table S7: Cases of LB in Eastern Europe from literature published from 2005 to 2020

Country	National or sub-national area	Author Year	Data source	Study Population	Study design	Study period	Case definition	Number of cases
Bulgaria [46]	Sofia	Baymakova 2016	Military Academy Hospital medical records	Patients with prolonged febrile syndrome	Retrospective cohort	2006-2010	LB	**2**
Bulgaria [47]	National	Deleva 2007	12 patients with LNB	NR	Prospective cohort	Not given	LNB: laboratory confirmed	**12**
Bulgaria [48]		Trifonova 2010	Serum samples	NR	Prospective cohort, Sero-epidemiological	2010	EM<br>Disseminated LB	**68**<br>**48**
Czech Republic [49]	Motol	Liba 2013	Hospital medical records	Pediatric neurology inpatients	Retrospective cohort	2009-2011	LNB: laboratory confirmed	**58**
Czech Republic [50]	National	Kriz 2017	National Register of Hospitalised Patients	National general population	Retrospective cross-sectional,	2003-2013	LB: ICD-10 codes	**23,631**
Czech Republic [51]	Prague	Palecek 2010	Medical records	Cardiology inpatients (N=39)	Prospective cross-sectional	2007-2008	LNB<br>LB detected in endocardial biopsy (PCR or electron microscopy)	**6392**<br>**8**
Hungary [53]	Budapest	Lakos 2010B	Center for Tick-borne Diseases, Budapest	Pregnant women	Retrospective observational	1986-2008	EM: CDC, EUCALB	**97**
Poland [54]	Podalskie	Moniuszko 2014	Medical Records, Department of Pediatric Orthopedics and Traumatology of the Medical University of Białystok.		Prospective cohort	2004-2010	LA, laboratory confirmed	**34**
Poland [55]	National	Moniuszko 2020	Serosurvey in patients with EM	Patients with EM	Prospective cohort, Sero-epidemiological	2012-2016	Clinical	310
Poland [56]	Krakow	Marek Kacinski 2007	Hospital-based data	NR	Prospective cohort	2005-2006	LNB: laboratory confirmed	**9**
Poland [57]	Bialystok	Krawczuk 2020	Medical records	Inpatients with LB	Retrospective cross-sectional	2004-2015	LNB: laboratory confirmed	**181**
Poland [58]	Kuyavian-Pomeranian Voivodship	Blazejewicz-Zawadziniska 2012	Local surveillance records	NR	Retrospective cohort	2000-2005	LB: confirmed by Western blot<br>EM	**973**<br>**825**

Country	National or sub-national area	Author Year	Data source	Study Population	Study design	Study period	Case definition	Number of cases
						2000	LB	**41**
						2001		**59**
						2002		**101**
						2003		**195**
						2004		**395**
						2005		**218**
						2000-2005	EM	**825**
							LA	**138**
							LNB	**53**
							LC	**2**
	Aleksandrowski					2000-2005	LB	**18**
	Brodnicki							**25**
	Bygoski							**433**
	Chełmiński							**10**
	Golubsko-dobrzyński							**15**
	Grudziądzki							**61**
	Inowrocławski							**37**
	Lipnowski							**3**
	Mogileński							**14**
	Nakielski							**48**
	Radziejowski							**1**
	Rypiński							**5**
	Sępóleński							**13**
	Świecki							**67**
	Toruński							**115**
	Tucholski							**51**
	Wąbrzeski							**19**
	Włocławski							**23**
	Zniński							**15**
Poland [59]	Białystok	Grygorczuk 2013	Medical records of patients with disseminated LB admitted to Department of Infectious Diseases & Neuroinfections	Patients with disseminated LB (N=33)	Retrospective observational	2012	LB	**33**
							LNB	**8**
							LA	**17**
							ACA	**5**
Poland [60]	West Pomeranian Province	Stawicki 2017	Retrospective analysis of	District sanitary-epidemiological	Retrospective cross-sectional	2005-2014	LB	**2756**

Country	National or sub-national area	Author Year	Data source	Study Population	Study design	Study period	Case definition	Number of cases
	Białogardzki<br>Choszczeński<br>Drawski<br>Goleniowski<br>Gryfice<br>Gryfiński<br>Kołobrzeski<br>Koszaliński<br>Policki<br>Pyrzycki<br>Stargardski<br>Szczecinek<br>City Szczecin<br>Świdwiński<br>City Swinoujscie<br>Wałecki<br>Mysliborski<br>Sławieński<br>Łobezki<br>Kamieński		surveillance mandatory notification data	stations (20 units) operating in the West Pomeranian Province				**59**<br>**73**<br>**131**<br>**124**<br>**146**<br>**182**<br>**109**<br>**139**<br>**155**<br>**19**<br>**60**<br>**106**<br>**761**<br>**79**<br>**140**<br>**248**<br>**47**<br>**43**<br>**40**<br>**95**
Poland [61]	Lublin region	Cisak 2008	Hospital medical records of clinical LB cases	Regional population.	Retrospective cohort	1998-2007	LB<br>LA<br>LNB<br>Skin borreliosis	**15**<br>**11**<br>**1**<br>**3**
Poland [6]	Podlaskie	Czupryna 2016	Medical records Department of Infectious Diseases and Neuro-infections, Medical Universty in Bialystok	Patients admitted with suspected LB (N=378)<br><br><br><br>Outpatients with EM	Retrospective cohort		LA: serological confirmation<br>LNB<br>LA<br>ACA<br>EM	**193**<br>**10**<br>**4**<br>**5**<br>**207**
Poland [62]	Lukow	Tokarska-Rodak 2010	Hospital-based data	Inpatients with suspected LB (N=200)	Prospective cohort	2007-2008	LB positive by Western blot	**100**
Romania [63]	County of Bihor	Constanta 2019	Retrospective clinical archives/records	Patients attending the Infectious Diseases hospital departments	Retrospective observational	2018-2019	LB	**132**
Romania [64]	Oradea	Andrei 2019	Emergency department medical records	NR	Prospective observational	2015-2018	EM with positive serology	**183**

Country	National or sub-national area	Author Year	Data source	Study Population	Study design	Study period	Case definition	Number of cases
Romania [65]	Cluj-Napoca	Lupse 2014	Hospital medical Records	NR	Prospective cohort	2011	EM: EUCALB	**44**
Romania [66]	Isai	Manciuc 2019	Hospital records	Patients with LB evaluated and treated at the hospital	Retrospective cohort	2017	LB: CDC	**89**
Romania [67]	Cluj-Napoca	Briciu 2013	Patient interview	Patients presenting to hospital with EM	Prospective observational	2011-2012	EM	**40**
Russia [68]	Moscow	Kritchevskaya 2014	Medical records fromtThe Helmholtz Eye Research Institute	Patients with inflammatory eye conditions	Observational	2013	Ophthalmic	**3**
Russia [69]	Altai region Siberian Federal district	Dedkov, 2017	Standardized questionnaire	Regional general population	Retrospective observational	2013-2015	IgM paired sera or IgG	**17**
Russia [70]	St. Petersburg	Grineva 2019	Children Clinical Research Center of Infectious Diseases	Children (1—17 yrs) with admitted with suspected LB	Prospective cohort	2010-2016	LB<br>EM	**184**<br>**142**
Slovak Republic [71]	Bratislava	Trnovcova 2007	Hospital-based data	Patients with EM	Prospective cohort	2001-2005	EM	**25**
Slovenia [72]	Ljubliana	Ogrinc 2016	Medical records University Medical Centre	Adult patients with a clinical diagnosis of Bannwarth syndrome attending the LB outpatient clinic	Prospective cohort	2005-2013	LNB: Bannwarth syndrome<br>EM	**77**<br>46
Slovenia [73]	Ljubliana	Maraspin 2019	Hospital medical records, University Medical Centre	Adults diagnosed with EM<br>Receiving treatment with tumor necrosis factor	Prospective cohort	2009-2018	 EM	**16**<br>**16**
Slovenia [74]	Ljubljana	Stupica 2011	LB Outpatient's Clinic, University Medical Center	Adults with EM and skin biopsy	Prospective cohort	2006	EM: CDC	**252**
Slovenia [75]	Ljubljana	Stupica 2015	LB Outpatient's Clinic, University Medical Center	Adults with EM and skin biopsy	Prospective cohort	2010	EM: EUCALB	**252**
Slovenia [76]	Ljubljana	Arnez 2011	Dept Infectious Disease,	Consecutive children (<15yrs) with EM	Prospective cohort	1996-2004	EM: CDC	**1164**

Country	National or sub-national area	Author Year	Data source	Study Population	Study design	Study period	Case definition	Number of cases
			University Medical Centre Ljubljana					
Slovenia [77]	Center Ljubljana	Maraspin 2015	Outpatient medical records	Adults with a hematological malignancy with EM	Prospective cohort	1992-2013	EM: EUCALB	**53**
				Immunocompetent controls				**106**
Ukraine [78]	National	Biletska 2008	Serological survey	Sick people with suspected LB	Prospective cohort	2003-2006	LB laboratory confirmed	**1650**

ACA, Acrodermatitis chronica atrophicans; CDC, the United States Centers for Disease Control and Prevention; CI, confidence interval; EM, erythema migrans; EUCALB, European Union Concerted Action on Lyme borreliosis; LA, Lyme arthritis; LB, Lyme borreliosis; LC, Lyme carditis; LNB, Lyme neuroborreliosis; NR, not reported

Table S8: Cases of LB in Nordic region from literature published from 2005 to 2020

Country	National or sub-national area	Author Year	Data source	Study Population	Study design	Study period	Case definition	Number of cases
Denmark [79]	National	Christiansen 2005	Surveillance data	Denmark population	Retrospective surveillance	2004<br>2005<br>1994-2004<br>2004-2005	LNB: laboratory confirmed<br><br>FP	**63**<br>**34**<br>**471**<br>**413**
Denmark [80]	National	Obel 2018	Danish laboratories and other national registries	Denmark population	Retrospective cohort	1985-2016	LNB: positive intrathecal antibody test	**2067**
Denmark [81]	East Zealand<br>North Zealand<br>Southwest Zealand<br>Funen<br>South Jutland<br>Mid Jutland<br>East Northwest Jutland<br>North Jutland<br>Bornholm	Tetens 2020	Danish national registries	Denmark population	Retrospective cohort	1996-2015	LNB: Positive intrathecal antibody test	**683**<br>**266**<br>**420**<br>**409**<br>**28**<br>**463**<br>**26**<br>**425**<br>**71**
Denmark [82]	Copenhagen, Funen, and North Jutland	Dessau 2010	GP survey of patients with a LB serology request	Regional populations	Cohort	2002-2003	LB: EUCALB<br>EM<br>LNB<br>ACA<br>LC<br>Chronic LNB<br>Lymphocytoma<br>Other<br>No current clinical symptoms	**2643**<br>**1011**<br>**340**<br>**67**<br>**14**<br>**130**<br>**28**<br>**330**<br>**367**
Denmark [83]	Eastern Denmark	Gynthersen 2020	233 referrals of the hospital Department of Infectious Disease with suspected LB	NR	Prospective cohort, Sero-epidemiological	2017-2019	LB: laboratory confirmed<br>EM<br>LNB<br>ACA<br>Multiple EM<br>LC<br>Post-treatment LB syndrome	**45**<br>**20**<br>**14**<br>**6**<br>**4**<br>**1**<br>**12**

Country	National or sub-national area	Author Year	Data source	Study Population	Study design	Study period	Case definition	Number of cases
Finland [84]	Helsinki	Kanerva 2013	Computerized search within the health-care district	Patients of Department of Otorhinolaryngology, Helsinki University Central Hospital	Prospective cohort	2007-2009	Intra-thecal antibodies and lymphocytic pleocytosis in CSF	**14**
Norway [85]	South-Rogaland district	Oymar 2009	Children admitted for possible LNB	Regional population	Retrospective cohort	1996-2006	LNB: laboratory confirmed	**143**
Norway [86]	High endemicity southern coastal areas	Ljostad 2007	Prospective hospital-based data	Adult > 18 years inpatients with LNB	Prospective cohort	2004-2006	LNB: Laboratory confirmed	**43**
Norway [87]	Agder county	Quarsten 2017	GP-recruited participants	70 adults with tick bite and symptoms	Prospective cohort, GP-based	2014-2015	EM<br>PCR positive	**65**<br>**3**
Norway [88]	National	Mysterud 2017	Surveillance data	Norway population	Retrospective cohort	1991-2015	Laboratory confirmed since 1995	**3424**
Norway [89]	South Rogaland district, Norway	Tveitnes 2007	Prospective data collection	Children <15 years admitted with facial palsy	Prospective cohort	1996-2004	CSF/antibody confirmation	**142**
Norway [90]	Vest-Agder	Ljostad-2005	Hospital based data	Adults admitted with facial palsy	Prospective cohort	1997-1998	CSF confirmed	**14**
Sweden [91]	Greater Stockholm	Bagger 2005	Children treated for LNB	Regional population	Prospective case-control	1995-1997	LNB	**255**
Sweden [92]	Stockholm	Arnason 2020	Hospital medical records	Children admitted with facial palsy	Retrospective cohort	2014-2015	LNB	**45**
Sweden [93]	Southern Sweden	Ornstein 2020	Prospective data	207 patients with EM and LNB	Prospective cohort	1994-1997	LB: CDC<br>LNB: CDC	**75**<br>**80**

ACA, Acrodermatitis chronica atrophicans; CDC, the United States Centers for Disease Control and Prevention; CI, confidence interval; EM, erythema migrans; EUCALB, European Union Concerted Action on Lyme borreliosis; LA, Lyme arthritis; LB, Lyme borreliosis; LC, Lyme carditis; LNB, Lyme neuroborreliosis; NR, not reported

Table S9: Cases of LB in the United Kingdom from literature published from 2005 to 2020

Country	National or sub-national area	Author Year	Data source	Study Population	Study design	Study period	Case definition	Number of cases
UK [94]	London	Dillon 2010	The Hospital for Tropical Diseases clinical database	Patients presenting to clinics	Prospective cohort	2002-2007	LB: Infectious Disease Society of America definition	**65**
UK [95]	South west England	Lovett 2008	Medical record	Patients with positive LB serology	Retrospective cohort	2000-2004	LB: serologically confirmed	**88**

ACA, Acrodermatitis chronica atrophicans; CDC, the United States Centers for Disease Control and Prevention; CI, confidence interval; EM, erythema migrans; EUCALB, European Union Concerted Action on Lyme borreliosis; LA, Lyme arthritis; LB, Lyme borreliosis; LC, Lyme carditis; LNB, Lyme neuroborreliosis; NR, not reported

Table S10: Cases of LB in Southern Europe from literature published from 2005 to 2020

Country	National or sub-national area	Author Year	Data source	Study Population	Study design	Study period	Case definition	Number of cases
Croatia [27]	Bjelovarsko-bilogorska county	Ropac 2013	Institute of Public health in Bjelovar-Bilogora, Croatia	NR	Retrospective cross-sectional	2007-2011	LB	**34**
Croatia [96]	National	Dzelalija 2015	Croatian National Institute of Public Health, Division of Epidemiology	Croatian national population	Retrospective cohort	2005	Clinical and epidemiology data, serology methods (IFA, ELISA), molecular (PCR), and culture	**220**
						2006		**301**
						2007		**266**
						2008		**439**
						2009		**435**
						2010		**492**
						2011		**499**
						2012		**434**
						2013		**661**
						2014		**470**
						2005-2014		**4217**
	Bjelovarsko-bilogorska					2005-2004		**77**
	Grad Zagreb							**1383**
	Koprivničko-križevačka							**204**
	Virovitičko-podravska							**88**
	Požeško-slavonska							**47**
	Brodsko-posavske							**70**
	Osječko-baranjska							**137**
	Vukovarsko-srijemska							**52**
	Sisa~ko-moslava~ko							**106**
	Karlovačka							**155**
	Primorsko-goranska							**292**
	Ličko-senjska							**5**
	Istarska							**72**
	Zadarska							**3**
	Šibensko-kninska							**0**
	Splitsko-dalmatinska							**13**
	Dubrovačko-neretvanska							**7**
	Međimurska							**274**
	Varaždinska							**207**
	Krapinsko zagorska							**427**
	Zagrebačka							**598**

Country	National or sub-national area	Author Year	Data source	Study Population	Study design	Study period	Case definition	Number of cases
Croatia [97]	National	Mulic 2006	Notifications to the Croatian Institute of Public Health	Croatian national population	Retrospective cohort	1988	LB	**4**
						1989		**4**
						1990		**1**
						1991		**168**
						1992		**96**
						1993		**306**
						1994		**260**
						1995		**274**
						1996		**335**
						1997		**229**
						1998		**248**
						1999		**232**
						2000		**235**
						2001		**313**
						2002		**292**
						2003		**326**
						1987-2003		**3317**
Italy [98]	National	Esposito 2021	Survey of 176 pediatric infectious disease physicians Registry of LD	Children with LB (N=160: 80 living in endemic and 80 in non-endemic areas)	Cross-sectional survey	2005-2011	EUCALB	**160**
	Endemic areas Friuli-Venezia Giulia, Trentino-Alto Adige, Liguria, Emilia-Romagna						EM	**66**
							LA	**10**
							LNB	**4**
	Non endemic areas: other regions of Italy						EM	**64**
							LA	**14**
							LNB	**2**
Italy [99]	Latium	Santino 2011	Laboratory records	Patients with serology data (N=954)	Retrospective cohort		LB: serologically confirmed	**42**
							EM	**21**
							LNB	**7**
							LA	**5**
Italy [100]	Northeast Italy	Marangoni 2008	Serology samples	Patients with EM	Retrospective cross-sectional, sero-epidemiological	NR	EM: Culture-confirmed	**66**
Turkey [101]	NR	Çelik 2016	Hospital medical records	Children with LNB	Retrospective cross-sectional,	2014-2015	LNB: serologically confirmed	**75**

ACA, Acrodermatitis chronica atrophicans; CDC, the United.States Centers for Disease Control and Prevention; CI, confidence interval; EM, erythema migrans; EUCALB, European Union Concerted Action on Lyme borreliosis; LA, Lyme arthritis; LB, Lyme borreliosis; LC, Lyme carditis; LNB, Lyme neuroborreliosis; NR, not reported

Table S11: Cases of LB in Central/Western Europe from literature published from 2005 to 2020

Country	National or sub-national area	Author Year	Data source	Study Population	Study design	Study period	Case definition	Number of cases
Belgium [102]	-	Linard 2007	Sentinel surveillance data, modelled estimates	Nationwide general population (adult)	Retrospective surveillance	1994	Laboratory confirmed cases	**~190**
						1995		**~200**
						1996		**~250**
						1997		**~320**
						1998		**~280**
						1999		**~380**
						2000		**~400**
						2001		**~550**
						2002		**~870**
						2003		**~700**
						2004		**~1400**
France [103]	National	Trombert 2005	Serosurvey in Pasteur-Cerba laboratory	Patients (N=1,330)	Retrospective cross-sectional, sero-epidemiological	2003	Serologically confirmed by Western blot	**425**
France [104]	National	Grillon 2019	The French National Reference Center for Borrelia database	France general population	Retrospective cohort	2010-2016	LA: Positive PCR in synovial fluid	**37**
France [105]	Besancon	Voitey 2020	Single medical center	Preferred for suspected LB	Retrospective cohort	2015-2018	LB: EUCALB	**48**
							LNB	**17**
							EM	**20**
							Early disseminated stage	**24**
							Other	**7**
France [106]	La Pitie Salpetriere Hospital, Paris	Kaminsky 2020	Hospital-based data	Patients with LNB	Prospective cohort	2007-2017	LNB: laboratory confirmed	**16**
France [107]	National	Jacquet 2019	Hospital medical records in Nancy	Patients referred to the hospital-based dedicated LB treatment pathway (N=468)	Retrospective cohort	2016-2017	LB: French Infectious Diseases Society	**69**
France [108]	Aquitaine, Limousin, Rhone-Alpes, Franche Comte, and Alsace	Mariet 2013	Sentinel GP surveillance network	Regional population	Prospective observational	2001-2012	EM: EUCALB	**1453**
	Aquitaine							**41**
	Limousin							**170**
	Rhone-Alpes							**335**

Country	National or sub-national area	Author Year	Data source	Study Population	Study design	Study period	Case definition	Number of cases
	Franche Comte<br>Alsace							**277**<br>**630**
Germany [109]	National	Lohr 2015	German statutory health insurance company (DAK-G)	Insured DAK-G population	Retrospective observational	2008-2011<br><br>2008<br>2009<br>2010<br>2011	LB (ICD-10 A69.2 + Code M01.2 arthritis	**2163**<br><br>**591**<br>**512**<br>**549**<br>**511**
Germany [110]	Munich	Buchholz 2014	Department of Neurology at Klinimuk Grosshadern, University of Munich	Inpatients with neuroinfectious diseases (N=376)	Retrospective cohort	2005-2009	LNB	**35**
The Netherlands [111]		Broekhuijsen-van Henten	Reports to the Dutch Paediatric Surveillance system	Children <18 y	Prospective cohort	2006-2007	LNB: EUCALB The Dutch Institute for Healthcare Improvement	**66**
The Netherlands [112]	Amsterdam	Nassar-2018	Medical records from the Department of Medical Microbiology and Academic Medical Centre	Children (< 18 years) with a clinical suspicion of LB, referred to the AMC in The Netherlands	Retrospective cohort	2011-2015	LB: Clinical Dx	**38**
Switzerland [113]	-	Monteventi 2018	The Swiss Neuropediatric Stroke Registry	Children with stroke	Prospective cohort<br>Retrospective	2000-2015	LNB: clinical and laboratory<br>Multiple EM	**4**<br><br>**545**
Switzerland [114]	Neuchatel	Huegli 2011	Questionnaire and blood samples	Residents presenting after tick bite in an endemic area (N=474)	Prospective cohort	2003-2005	LB confirmed by Western blot<br>EM	**12**<br><br>**14**

ACA, Acrodermatitis chronica atrophicans; CDC, the United.States Centers for Disease Control and Prevention; EM, erythema migrans; EUCALB, European Union Concerted Action on Lyme borreliosis; LA, Lyme arthritis; LB, Lyme borreliosis; LC, Lyme carditis; LNB, Lyme neuroborreliosis; NR, not reported

Footnote for all Supplement Tables

Incidence estimates and 95% CIs have been rounded to the first decimal to the right for consistent level of precision.

References

[1] Daniel M, Kříz˘ B, Valter J, Kott I, Danielová V. The influence of meteorological conditions of the preceding winter on the incidences of tick-borne encephalitis and Lyme borreliosis in the Czech Republic. Int J Med Microbiol 2008;298:60-7. https://dx.doi.org/10.1016/j.ijmm.2008.05.001.

[2] Toczylowski K, Bojkiewicz E, Barszcz M, Wozinska-Klepadlo M, Potocka P, Sulik A. Etiology, Clinical Presentation and Incidence of Infectious Meningitis and Encephalitis in Polish Children. J Clin Med 2020;9: https://dx.doi.org/10.3390/jcm9082324.

[3] Zbrzeźniak J, Paradowska-Stankiewicz I. Lyme disease in Poland in 2017. Przegl Epidemiol 2019;73:317-20. https://dx.doi.org/10.32394/pe.73.37.

[4] Paradowska-Stankiewicz I. Lyme disease in Poland in 2013. Przegl Epidemiol 2015;69:763-4 (En), 897-8 (Pl).

[5] Krzyżak M, Paszko A, Filipkowska M, Maślach D. Lyme borreliosis in Podlaskie Province in 2011-2018 – trend analysis. Medycyna Ogólna i Nauki o Zdrowiu 2019;25:235-9. https://dx.doi.org/10.26444/monz/114443.

[6] Czupryna P, Moniuszko-Malinowska A, Pancewicz S, Garkowski A, Goscik J, Siemieniako A, et al. Lyme disease in Poland - A serious problem? Adv Med Sci 2016;61:96-100. https://dx.doi.org/10.1016/j.advms.2015.10.007.

[7] Stefanoff P, Rosińska M, Zieliński A. Epidemiology of tick-borne diseases in Poland. Przegl Epidemiol 2006;60:151-9.

[8] Ţilea B, Tripon G, Voidăzan S, Ţilea I. Serological, clinical and epidemiological aspects of Lyme borreliosis in Mures County, Romania. Revista Română de Medicină de Laborator 2014;22:233-44.

[9] Bogachkina S, Rakitin I, Meltser A, Parkov O, Chhindzheria I, Antyikova L, et al. Importance of acaricide treatment for prevention of natural focal infections of tick-borne encephalitis and Lyme borreliosis in St. Petersburg. EpiNorth - Journal of the Network for Communicable Disease Control in Northern and Eastern Europe 2011;12:76-81.

[10] Bochničková M, Szilágyiová M, Gardlík R. Lyme borreliosis - epidemiological analysis of incidence in the northern region of Slovakia. Epidemiologie, Mikrobiologie, Imunologie 2012;61:3-8.

[11] Petrulioniene A, Radzisauskiene D, Paulauskas A, Venalis A. Lyme Disease among Patients at an Ambulatory Unit in a Highly Endemic Country: Lithuania. Medicina (Kaunas) 2021;57: https://dx.doi.org/10.3390/medicina57020184.

[12] Dessau RB, Espenhain L, Mølbak K, Krause TG, Voldstedlund M. Improving national surveillance of Lyme neuroborreliosis in Denmark through electronic reporting of specific antibody index testing from 2010 to 2012. Eurosurveillance 2015;20:21184.

[13] Andreasen AM, Dehlendorff PB, Knudtzen FC, Bødker R, Kjær LJ, Skarphedinsson S. Spatial and temporal patterns of Lyme Neuroborreliosis on Funen, Denmark from 1995-2014. Sci Rep 2020;10:7796.

[14] Knudtzen FC, Andersen NS, Jensen TG, Skarphédinsson S. Characteristics and clinical outcome of Lyme neuroborreliosis in a high endemic area, 1995-2014: a retrospective cohort study in Denmark. Clin Infect Dis 2017;65:1489-95.

[15] Krabbe NV, Jensen TE, Nielsen HI. [Neuroborreliosis in North Jutland]. Ugeskr Laeger 2008;170:3420-4.

[16] Sajanti E, Virtanen M, Helve O, Kuusi M, Lyytikäinen O, Hytönen J, et al. Lyme borreliosis in Finland, 1995-2014. Emerg Infect Dis 2017;23:1282-8. https://dx.doi.org/10.3201/eid2308.161273.

[17] Tveitnes D, Natås OB, Skadberg Ø, Øymar K. Lyme meningitis, the major cause of childhood meningitis in an endemic area: a population based study. Arch Dis Child 2012;97:215-20.

[18] Eliassen KE, Berild D, Reiso H, Grude N, Christophersen KS, Finckenhagen C, et al. Incidence and antibiotic treatment of erythema migrans in Norway 2005-2009. Ticks Tick Borne Dis 2017;8:1-8. https://dx.doi.org/10.1016/j.ttbdis.2016.06.006.

[19] Bennet L, Halling A, Berglund J. Increased incidence of Lyme borreliosis in southern Sweden following mild winters and during warm, humid summers. European Journal of Clinical

Microbiology & Infectious Diseases 2006;25:426-32. https://dx.doi.org/10.1007/s10096-006-0167-2.
[20] Sodermark L, Sigurdsson V, Nas W, Wall P, Trollfors B. Neuroborreliosis in Swedish Children: A Population-based Study on Incidence and Clinical Characteristics. Pediatr Infect Dis J 2017;36:1052-6. https://dx.doi.org/10.1097/INF.0000000000001653.
[21] Vellinga A, Kilkelly H, Cullinan J, Hanahoe B, Cormican M. Geographic distribution and incidence of Lyme borreliosis in the west of Ireland. Irish Journal of Medical Science 2018;187:435-40.
[22] Mavin S, Watson EJ, Evans R. Distribution and presentation of Lyme borreliosis in Scotland - analysis of data from a national testing laboratory. J R Coll Physicians Edinb 2015;45:196-200. https://dx.doi.org/10.4997/JRCPE.2015.304.
[23] Milner RM, Mavin S, Ho-Yen DO. Lyme borreliosis in Scotland during two peak periods. J R Coll Physicians Edinb 2009;39:196-9.
[24] Slack GS, Mavin S, Yirrell D, Ho-Yen DO. Is Tayside becoming a Scottish hotspot for Lyme borreliosis? J R Coll Physicians Edinb 2011;41:5-8. https://dx.doi.org/10.4997/JRCPE.2011.102.
[25] Cairns V, Wallenhorst C, Rietbrock S, Martinez C. Incidence of Lyme disease in the UK: a population-based cohort study. BMJ Open 2019;9:e025916. https://dx.doi.org/10.1136/bmjopen-2018-025916.
[26] Mulić R, Petković B, Klišmanić Z, Jerončić I. Tick-borne diseases in the Republic of Croatia. Lijecnicki Vjesnik 2011;133:89-95.
[27] Ropac D, Holubek M, Grgić V, Srašević I. Lyme disease in the area of Bjelovarsko-bilogorska County - negligible or neglected disease. Croatian Journal of Infection 2013;33:21-6.
[28] Zanzani SA, Rimoldi SG, Manfredi M, Grande R, Gazzonis AL, Merli S, et al. Lyme borreliosis incidence in Lombardy, Italy (2000-2015): Spatiotemporal analysis and environmental risk factors. Ticks Tick Borne Dis 2019;10:101257. https://dx.doi.org/10.1016/j.ttbdis.2019.07.001.
[29] Vázquez-López ME, Pego-Reigosa R, Díez-Morrondo C, Castro-Gago M, Díaz P, Fernández G, et al. Epidemiology of Lyme disease in a healthcare area in north-west Spain. Gac Sanit 2015;29:213-6. https://dx.doi.org/10.1016/j.gaceta.2015.01.008.
[30] Vázquez-López ME, Díez-Morrondo C, Sánchez-Andrade A, Pego-Reigosa R, Díaz P, Castro-Gago M. Articular manifestations in patients with Lyme disease. Reumatol Clin 2016;12:327-30.
[31] Vazquez-Lopez ME, Perez-Pacin R, Diez-Morrondo C, Diaz P, Castro-Gago M. [Lyme disease in paediatrics]. An Pediatr (Barc) 2016;84:234-5. https://dx.doi.org/10.1016/j.anpedi.2015.07.008.
[32] Geebelen L, Van Cauteren D, Devleesschauwer B, Moreels S, Tersago K, Van Oyen H, et al. Combining primary care surveillance and a meta-analysis to estimate the incidence of the clinical manifestations of Lyme borreliosis in Belgium, 2015-2017. Ticks Tick Borne Dis 2019;10:598-605. https://dx.doi.org/10.1016/j.ttbdis.2018.12.007.
[33] Raguet S, Strat Yl, Chouin L, Hansmann Y, Martinot M, Kieffer P, et al. Incidence of Lyme borreliosis in the Alsace districts: alsa(ce)tique study 2014-2015, France. Bulletin Épidémiologique Hebdomadaire 2018;406-12.
[34] Septfons A, Goronflot T, Jaulhac B, Roussel V, De Martino S, Guerreiro S, et al. Epidemiology of Lyme borreliosis through two surveillance systems: the national Sentinelles GP network and the national hospital discharge database, France, 2005 to 2016. Eurosurveillance 2019;24: https://dx.doi.org/10.2807/1560-7917.es.2019.24.11.1800134.
[35] Beytout J, George JC, Malaval J, Garnier M, Beytout M, Baranton G, et al. Lyme borreliosis incidence in two French departments: correlation with infection of Ixodes ricinus ticks by Borrelia burgdorferi sensu lato. Vector Borne Zoonotic Dis 2007;7:507-17. https://dx.doi.org/10.1089/vbz.2006.0633.
[36] Tessier S, Strat Yl, Serre A, Leroy J, Martino Sd, Jaulhac B, et al. Study on Lyme borreliosis and six others tick-borne diseases, Franche-Comté (France), 2010-2012. Bulletin Épidémiologique Hebdomadaire 2018;413-8.

[37] Schmitt M, Encrenaz N, Chubilleau C, Verrier A. Epidemiological data on Lyme disease in Alsace, Limousin and Rhône-Alpes. Bulletin Épidémiologique Hebdomadaire 2006;202-3.
[38] Letrilliart L, Ragon B, Hanslik T, Flahault A. Lyme disease in France: a primary care-based prospective study. Epidemiology and Infection 2005;133:935-42.
[39] Fulop B, Poggensee G. Epidemiological situation of Lyme borreliosis in germany: surveillance data from six Eastern German States, 2002 to 2006. Parasitol Res 2008;103 Suppl 1:S117-20. https://dx.doi.org/10.1007/s00436-008-1060-y.
[40] Heinzinger S, Böhmer M, Belting A, Liebl B, Wildner M, Sing A, et al. LYDI-Sentinel and mandatory reporting of Lyme borreliosis monitoring in Bavaria from 2013 to 2016. 2017;300-3.
[41] Mehnert WH, Krause G. [HTML] Surveillance of Lyme borreliosis in Germany, 2002 and 2003. Euro surveillance : bulletin européen sur les maladies transmissibles = European communicable disease bulletin 2005;10:83-5.
[42] Adlhoch C, Poggensee G. Lyme-Borreliose: Ein Situationsbericht aus den sechs östlichen Bundesländern 2007–2009. In: Editor, editor.^editors. Book Lyme-Borreliose: Ein Situationsbericht aus den sechs östlichen Bundesländern 2007–2009; 2010: p. 5-8.
[43] Bierman SM, van Kooten B, Vermeeren YM, Bruintjes TD, van Hees BC, Bruinsma RA, et al. Incidence and characteristics of lyme neuroborreliosis in adult patients with facial palsy in an endemic area in the Netherlands. Epidemiol Infect 2019;147:1-6.
[44] Botman E, Ang CW, Joosten JHK, Slottje P, van der Wouden JC, Maarsingh OR. Diagnostic behaviour of general practitioners when suspecting Lyme disease: a database study from 2010-2015. BMC family practice 2018;19:43.
[45] Altpeter E, Zimmermann H, Oberreich J, Peter O, Dvorak C, Swiss Sentinel Surveillance N. Tick related diseases in Switzerland, 2008 to 2011. Swiss Med Wkly 2013;143:w13725. https://dx.doi.org/10.4414/smw.2013.13725.
[46] Baymakova M, Plochev K, Dikov I, Kundurdjiev T, Popov GT, Kovaleva V. The prevalence of vector-borne diseases among patients with fever of unknown origin in a Bulgarian hospital. Archives of Hellenic Medicine 2016;33:656-60.
[47] Deleva N, Kaprelyan A, Geneva S, Tzoukeva A, Dimitrov I. Cranial and spinal neuropathy in lyme neuroborreliosis. Journal of IMAB - Annual Proceeding (Scientific Papers) 2007;13:54-6.
[48] Trifonova I, Gladnishka T, Tasseva E, Ivanova V, Christova I. Human serum reactivity against Borrelia OspC, OspA, FlaB and VlsE protein antigens in early and disseminated Lyme borreliosis. Probl Inf Parasit Dis 2010;38:11-4.
[49] Libá-Vrabelová Z, Kayserová J, Komárek V. Frequent incidence of lyme neuroborreliosis in children in the Czech Republic. Ceska a Slovenska Neurologie a Neurochirurgie 2013;76:63-9.
[50] Kříž B, Malý M, Daniel M. Neuroborreliosis in patients hospitalised for Lyme borreliosis in the Czech Republic in 2003 - 2013. Epidemiol Mikrobiol Imunol 2017;66:115-23.
[51] Palecek T, Kuchynka P, Hulinska D, Schramlova J, Hrbackova H, Vitkova I, et al. Presence of Borrelia burgdorferi in endomyocardial biopsies in patients with new-onset unexplained dilated cardiomyopathy. Med Microbiol Immunol 2010;199:139-43. https://dx.doi.org/10.1007/s00430-009-0141-6.
[52] !!! INVALID CITATION !!! [55];
[53] Lakos A, Solymosi N. Maternal Lyme borreliosis and pregnancy outcome. Int J Infect Dis 2010;14:e494-e8.
[54] Moniuszko A, Popko J, Guszczyn T, Waliński T, Żukiewicz-Sobczak W, Pancewicz S. Lyme disease with effusion either in hip or knee in children from Podlaskie region treated in clinic in 2004-2010. Przegl Epidemiol 2014;68:425-8, 535-7 (Pl).
[55] Moniuszko-Malinowska A, Dunaj J, Andersson MO, Czupryna P, Zajkowska J, Guziejko K, et al. Assessment of Anaplasma phagocytophilum presence in early Lyme borreliosis manifested by erythema migrans skin lesions. Travel Med Infect Dis 2020;36:101648. https://dx.doi.org/10.1016/j.tmaid.2020.101648.
[56] Kaciński M, Zajac A, Skowronek-Bała B, Kroczka S, Gergont A, Kubik A. CNS Lyme disease manifestation in children. Przegląd lekarski 2007;64:38-40.

[57] Krawczuk K, Czupryna P, Pancewicz S, Oldak E, Krol M, Moniuszko-Malinowska A. Comparison of Neuroborreliosis Between Children and Adults. Pediatr Infect Dis J 2020;39:7-11. https://dx.doi.org/10.1097/INF.0000000000002493.
[58] Błazejewicz-Zawadzińiska M, Brochocka A, Lisińska J, Borowiecki M. [A reprospective analysis of 973 patients with lyme borreliosis in Kuyavian-Pomeranian voivodship in 2000-2005]. Przegląd epidemiologiczny 2012;66:581-6.
[59] Grygorczuk S, Peter O, Kondrusik M, Moniuszko A, Zajkowska J, Dunaj J, et al. Assessment of the frequency of different Borrelia burgdorferi sensu lato species in patients with Lyme borreliosis from north-east Poland by studying preferential serologic response and DNA isolates. Ann Agric Environ Med 2013;20:21-9.
[60] Stawicki T. Studies on Lyme disease incidence rates in selected groups of forestry workers in West Pomerania, 2005-2014. Med Pr 2017;68:211-20.
[61] Cisak E, Chmielewska-Badora J, Zwoliñski J, Wójcik-Fatla A, Zając V, Skórska C, et al. Study on Lyme borreliosis focus in the Lublin Region (Eastern Poland). Annals of Agricultural and Environmental Medicine 2008;15:327-32.
[62] Tokarska-Rodak M, Fota-Markowska H, Śmiechowicz F, Gajownik B, Prokop M, Modrzewska R, et al. Antibodies against in vivo B. burgdorferi antigens evaluated in patients with lyme arthritis with reference to treatment. Advances in Clinical and Experimental Medicine 2010;19:489-96.
[63] Constanta T, Veronica H. Incidence of Lyme disease in Bihor county in 2018-2019 in compared with 2012-2014. Analele Universitatii din Oradea, Fascicula: Ecotoxicologie, Zootehnie si Tehnologii de Industrie Alimentara 2019;18:293-303.
[64] Andrei C. Epidemiological and clinical issues of patients with thick bites and first stage Lyme disease, in Bihor county. Analele Universitatii din Oradea, Fascicula: Ecotoxicologie, Zootehnie si Tehnologii de Industrie Alimentara 2019;18:261-6.
[65] Lupșe M, Briciu V, Flonta M, Nastase V, Todor N, Kullberg B-J. Serological and Clinical one year follow-up of Patients with Erythema migrans treated in a Romanian Infectious Disease Hospital. Romanian Review of Laboratory Medicine 2014;22: https://dx.doi.org/10.2478/rrlm-2014-0021.
[66] Manciuc C, Vata A, Filip-Ciubotaru F, Luca MC, Largu A, Iordan IF. Environmental changes in North-Eastern Romania - a trigger factor for Lyme disease. Environmental Engineering and Management Journal 2019;18:775-9.
[67] Briciu VT, Țățulescu DF, Flonta MMM, Năstase V, Cârstina D, Lupșe MS. Tick bites and Erythema migrans in Transylvania. Scientia Parasitologica 2013;14:21-9.
[68] Kritchevskaya GI, Vakhova ES, Vylivannaya OB, Davydova GA. The rate of detection and diagnostic significance of antibodies to borrelia burgdorferri in patients with eyes diseases of inflammatory nature. Klinichescheskaya Laboratornaya Diagnostika 2014;53-6.
[69] Dedkov VG, Simonova EG, Beshlebova OV, Safonova MV, Stukolova OA, Verigina EV, et al. The burden of tick-borne diseases in the Altai region of Russia. Ticks Tick Borne Dis 2017;8:787-94. https://dx.doi.org/10.1016/j.ttbdis.2017.06.004.
[70] Grineva AA, Skripchenko NV. Clinical and Pathogenetic Peculiarities of Borreliosis Infection in Children. Pediatria Journal named after GN Speransky 2019;98:45-52. https://dx.doi.org/10.24110/0031-403x-2019-98-1-45-52.
[71] Trnovcova M, Bazovska S, Svecova D. Antibodies to Borrelia burgdorferi in erythema migrans patients. Bratisl Lek Listy 2007;108:399-402.
[72] Ogrinc K, Lusa L, Lotrič-Furlan S, Bogovič P, Stupica D, Cerar T, et al. Course and outcome of early European lyme neuroborreliosis (Bannwarth syndrome): clinical and Laboratory findings. Clin Infect Dis 2016;63:346-53.
[73] Maraspin V, Bogovic P, Rojko T, Ogrinc K, Ruzic-Sabljic E, Strle F. Early Lyme Borreliosis in Patients Treated with Tumour Necrosis Factor-Alfa Inhibitors. J Clin Med 2019;8: https://dx.doi.org/10.3390/jcm8111857.
[74] Stupica D, Lusa L, Cerar T, Ruzic-Sabljic E, Strle F. Comparison of post-Lyme Borreliosis symptoms in erythema migrans patients with positive and negative Borrelia burgdorferi sensu lato skin culture. Vector Borne Zoonotic Dis 2011;11:883-9. https://dx.doi.org/10.1089/vbz.2010.0018.

[75] Stupica D, Lusa L, Maraspin V, Bogovič P, Vidmar D, O'Rourke M, et al. Correlation of culture positivity, PCR positivity, and burden of Borrelia burgdorferi sensu lato in skin samples of erythema migrans patients with clinical findings. PLoS One 2015;10:e0136600.
[76] Arnež M, Ružić-Sabljić E. Borrelia burgdorferi sensu lato bacteremia in Slovenian children with solitary and multiple erythema migrans. Pediatr Infect Dis J 2011;30:988-99.
[77] Maraspin V, Ružić-Sabljić E, Lusa L, Strle F. Course and outcome of early Lyme borreliosis in patients with hematological malignancies. Clin Infect Dis 2015;61:427-31.
[78] Biletska H, Podavalenko L, Semenyshyn O, Lozynskyj I, Tarasyuk O. Study of Lyme borreliosis in Ukraine. International Journal of Medical Microbiology 2008;298:154-60. https://dx.doi.org/10.1016/j.ijmm.2008.04.004.
[79] Christiansen A, Mølbak K. Neuroborreliose 1994-2004. Statens Serum Institut. EPI-NYT17 August 2005.
[80] Obel N, Dessau RB, Krogfelt KA, Bodilsen J, Andersen NS, Moller JK, et al. Long term survival, health, social functioning, and education in patients with European Lyme neuroborreliosis: nationwide population based cohort study. BMJ 2018;361:k1998. https://dx.doi.org/10.1136/bmj.k1998.
[81] Tetens MM, Haahr R, Dessau RB, Krogfelt KA, Bodilsen J, Andersen NS, et al. Changes in Lyme neuroborreliosis incidence in Denmark, 1996 to 2015. Ticks Tick Borne Dis 2020;11:101549. https://dx.doi.org/10.1016/j.ttbdis.2020.101549.
[82] Dessau RB, Bangsborg JM, Ejlertsen T, Skarphedinsson S, Schønheyder HC. Utilization of serology for the diagnosis of suspected Lyme borreliosis in Denmark: survey of patients seen in general practice. BMC Infect Dis 2010;10:(1 November 2010).
[83] Gynthersen RMM, Tetens MM, Orbaek M, Haahr R, Fana V, Hansen K, et al. Classification of patients referred under suspicion of tick-borne diseases, Copenhagen, Denmark. Ticks Tick Borne Dis 2021;12:101591. https://dx.doi.org/10.1016/j.ttbdis.2020.101591.
[84] Kanerva M, Nissinen J, Moilanen K, Mäki M, Lahdenne P, Pitkäranta A. Microbiologic findings in acute facial palsy in children. Otology & neurotology : official publication of the American Otological Society, American Neurotology Society [and] European Academy of Otology and Neurotology 2013;34:e82-7.
[85] Oymar K, Tveitnes D. Clinical characteristics of childhood Lyme neuroborreliosis in an endemic area of northern Europe. Scand J Infect Dis 2009;41:88-94. https://dx.doi.org/10.1080/00365540802593453.
[86] Ljostad U, Skarpaas T, Mygland A. Clinical usefulness of intrathecal antibody testing in acute Lyme neuroborreliosis. Eur J Neurol 2007;14:873-6. https://dx.doi.org/10.1111/j.1468-1331.2007.01799.x.
[87] Quarsten H, Grankvist A, Hoyvoll L, Myre IB, Skarpaas T, Kjelland V, et al. Candidatus Neoehrlichia mikurensis and Borrelia burgdorferi sensu lato detected in the blood of Norwegian patients with erythema migrans. Ticks Tick Borne Dis 2017;8:715-20. https://dx.doi.org/10.1016/j.ttbdis.2017.05.004.
[88] Mysterud A, Jore S, Osteras O, Viljugrein H. Emergence of tick-borne diseases at northern latitudes in Europe: a comparative approach. Sci Rep 2017;7:16316. https://dx.doi.org/10.1038/s41598-017-15742-6.
[89] Tveitnes D, Oymar K, Natas O. Acute facial nerve palsy in children: how often is it lyme borreliosis? Scand J Infect Dis 2007;39:425-31. https://dx.doi.org/10.1080/00365540601105764.
[90] Ljostad U, Okstad S, Topstad T, Mygland A, Monstad P. Acute peripheral facial palsy in adults. J Neurol 2005;252:672-6. https://dx.doi.org/10.1007/s00415-005-0715-1.
[91] Bagger-Sjoback D, Remahl S, Ericsson M. Long-term outcome of facial palsy in neuroborreliosis. Otol Neurotol 2005;26:790-5. https://dx.doi.org/10.1097/01.mao.0000178148.98762.00.
[92] Arnason S, Hultcrantz M, Nilsson A, Laestadius A. Peripheral facial nerve palsy in children in a Borrelia high-endemic area, a retrospective follow-up study. Acta Paediatr 2020;109:1229-35. https://dx.doi.org/10.1111/apa.15063.
[93] Ornstein K, Berglund J, Nilsson I, Norrby R, Bergstrom S. Characterization of Lyme borreliosis isolates from patients with erythema migrans and neuroborreliosis in southern

Sweden. J Clin Microbiol 2001;39:1294-8. https://dx.doi.org/10.1128/JCM.39.4.1294-1298.2001.
[94] Dillon R, O'Connell S, Wright S. Lyme disease in the UK: Clinical and laboratory features and response to treatment. Clinical Medicine, Journal of the Royal College of Physicians of London 2010;10:454-7.
[95] Lovett JK, Evans PH, O'Connell S, Gutowski NJ. Neuroborreliosis in the South West of England. Epidemiol Infect 2008;136:1707-11.
[96] Dželalija B, Medić A, Novosel IP, Sablić S. Zoonoses in the Republic of Croatia. Croatian Journal of Infection 2015;35:45-51.
[97] Mulić R, Antonijević S, Klišmanić Z, Ropac D, Lučev O. Epidemiological characteristics and clinical manifestations of Lyme borreliosis in Croatia. Mil Med 2006;171:1105-9.
[98] Esposito S, Baggi E, Villani A, Norbedo S, Pellegrini G, Bozzola E, et al. Management of paediatric Lyme disease in non-endemic and endemic areas: data from the Registry of the Italian Society for Pediatric Infectious Diseases. Eur J Clin Microbiol Infect Dis 2013;32:523-9. https://dx.doi.org/10.1007/s10096-012-1768-6.
[99] Santino I, Longobardi V. Clinical and serological features of patients with suspected lyme borreliosis. Int J Immunopathol Pharmacol 2011;24:797-801.
[100] Marangoni A, Moroni A, Accardo S, Cevenini R. Borrelia burgdorferi VlsE antigen for the serological diagnosis of Lyme borreliosis. Eur J Clin Microbiol Infect Dis 2008;27:349-54. https://dx.doi.org/10.1007/s10096-007-0445-7.
[101] Celik T, Celik U, Kömür M, Tolunay O, Yildizdas RD, Yagci-Kupeli B, et al. Pediatric Lyme Neuroborreliosis: Different clinical presentations of the same agent; Single center experience. Neuro Endocrinol Lett 2016;37:107-13.
[102] Linard C, Lamarque P, Heyman P, Ducoffre G, Luyasu V, Tersago K, et al. Determinants of the geographic distribution of Puumala virus and Lyme borreliosis infections in Belgium. Int J Health Geogr 2007;6:15. https://dx.doi.org/10.1186/1476-072X-6-15.
[103] Trombert-Paolantoni S, Ferquel E, Postic D. [Retrospective study of Lyme borreliosis positive serologies in 2003]. Pathol Biol (Paris) 2005;53:522-6. https://dx.doi.org/10.1016/j.patbio.2005.06.010.
[104] Grillon A, Scherlinger M, Boyer PH, De Martino S, Perdriger A, Blasquez A, et al. Characteristics and clinical outcomes after treatment of a national cohort of PCR-positive Lyme arthritis. Semin Arthritis Rheum 2019;48:1105-12.
[105] Voitey M, Bouiller K, Chirouze C, Fournier D, Bozon F, Klopfenstein T. Functional signs in patients consulting for presumed Lyme borreliosis. Med Mal Infect 2020;50:423-7. https://dx.doi.org/10.1016/j.medmal.2019.10.011.
[106] Kaminsky AL, Maisonobe T, Lenglet T, Psimaras D, Debs R, Viala K. Confirmed cases of Neuroborreliosis with involvement of peripheral nervous system: Description of a cohort. Medicine 2020;99:e21986.
[107] Jacquet C, Goehringer F, Baux E, Conrad JA, Ganne Devonec MO, Schmutz JL, et al. Multidisciplinary management of patients presenting with Lyme disease suspicion. Med Mal Infect 2019;49:112-20. https://dx.doi.org/10.1016/j.medmal.2018.06.002.
[108] Mariet AS, Retel O, Avocat H, Serre A, Schapman L, Schmitt M, et al. Estimated incidence of erythema migrans in five regions of France and ecological correlations with environmental characteristics. Vector Borne Zoonotic Dis 2013;13:666-73. https://dx.doi.org/10.1089/vbz.2012.1199.
[109] Lohr B, Muller I, Mai M, Norris DE, Schoffski O, Hunfeld KP. Epidemiology and cost of hospital care for Lyme borreliosis in Germany: lessons from a health care utilization database analysis. Ticks Tick Borne Dis 2015;6:56-62. https://dx.doi.org/10.1016/j.ttbdis.2014.09.004.
[110] Buchholz G, Ormanns S, Pfister HW, Koedel U, Klein M. Neuroinfectious diseases at a European neurological tertiary care center: one-third of patients require treatment in the neurological intensive care unit. Eur J Neurol 2014;21:1500-3. https://dx.doi.org/10.1111/ene.12379.
[111] Broekhuijsen-van Henten DM, Braun KP, Wolfs TF. Clinical presentation of childhood neuroborreliosis; neurological examination may be normal. Arch Dis Child 2010;95:910-4. https://dx.doi.org/10.1136/adc.2009.176529.

[112] Nassar-Sheikh Rashid A, Boele Van Hensbroek M, Kolader M, Hovius JW, Pajkrt D. Lyme borreliosis in children: A tertiary referral hospital-based retrospective analysis. Pediatr Infect Dis J 2018;37:e45-e7.

[113] Monteventi O, Steinlin M, Regényi M, Roulet-Perez E, Weber P, Fluss J. Pediatric stroke related to Lyme neuroborreliosis: Data from the Swiss NeuroPaediatric Stroke Registry and literature review. Eur J Paediatr Neurol 2018;22:113-21.

[114] Huegli D, Moret J, Rais O, Moosmann Y, Erard P, Malinverni R, et al. Prospective study on the incidence of infection by Borrelia burgdorferi sensu lato after a tick bite in a highly endemic area of Switzerland. Ticks Tick Borne Dis 2011;2:129-36. https://dx.doi.org/10.1016/j.ttbdis.2011.05.002.

[116] Wilking H, Stark K. Trends in surveillance data of human Lyme borreliosis from six federal states in eastern Germany, 2009-2012. *Ticks Tick Borne Dis*. 2014;5(3):219-224. doi:10.1016/j.ttbdis.2013.10.010
